# Rescue of premature aging defects in Cockayne syndrome stem cells by CRISPR/Cas9-mediated gene correction

**DOI:** 10.1007/s13238-019-0623-2

**Published:** 2019-04-30

**Authors:** Si Wang, Zheying Min, Qianzhao Ji, Lingling Geng, Yao Su, Zunpeng Liu, Huifang Hu, Lixia Wang, Weiqi Zhang, Keiichiro Suzuiki, Yu Huang, Puyao Zhang, Tie-Shan Tang, Jing Qu, Yang Yu, Guang-Hui Liu, Jie Qiao

**Affiliations:** 1 Department of Obstetrics and Gynecology, Center for Reproductive Medicine, Peking University Third Hospital, Beijing, 100191 China; 2 National Laboratory of Biomacromolecules, CAS Center for Excellence in Biomacromolecules, Institute of Biophysics, Chinese Academy of Sciences, Beijing, 100101 China; 3 State Key Laboratory of Stem Cell and Reproductive Biology, Institute of Zoology, Chinese Academy of Sciences, Beijing, 100101 China; 4 University of Chinese Academy of Sciences, Beijing, 100049 China; 5 Advanced Innovation Center for Human Brain Protection, National Clinical Research Center for Geriatric Disorders, Xuanwu Hospital Capital Medical University, Beijing, 100053 China; 6 Institute for Stem cell and Regeneration, Chinese Academy of Sciences, Beijing, 100101 China; 7 Key Laboratory of Genomic and Precision Medicine, Beijing Institute of Genomics, Chinese Academy of Sciences, Beijing, 100101 China; 8 Beijing Institute for Brain Disorders, Beijing, 100069 China; 9 Institute for Advanced Co-Creation Studies, Osaka University, Osaka, 560-8531 Japan; 10 Graduate School of Engineering Science, Osaka University, Osaka, 560-8531 Japan; 11 Department of Medical Genetics, School of Basic Medical Sciences, Peking University Health Science Center, Beijing, 100191 China; 12 State Key Laboratory of Membrane Biology, Institute of Zoology, Chinese Academy of Sciences, Beijing, 100101 China; 13 Peking-Tsinghua Center for Life Sciences, Academy for Advanced Interdisciplinary Studies, Peking University, Beijing, 100871 China

**Keywords:** Cockayne syndrome, CRISPR/Cas9, gene correction, disease modelling, mesenchymal stem cell, neural stem cell

## Abstract

Cockayne syndrome (CS) is a rare autosomal recessive inherited disorder characterized by a variety of clinical features, including increased sensitivity to sunlight, progressive neurological abnormalities, and the appearance of premature aging. However, the pathogenesis of CS remains unclear due to the limitations of current disease models. Here, we generate integration-free induced pluripotent stem cells (iPSCs) from fibroblasts from a CS patient bearing mutations in *CSB*/*ERCC6* gene and further derive isogenic gene-corrected CS-iPSCs (GC-iPSCs) using the CRISPR/Cas9 system. CS-associated phenotypic defects are recapitulated in CS-iPSC-derived mesenchymal stem cells (MSCs) and neural stem cells (NSCs), both of which display increased susceptibility to DNA damage stress. Premature aging defects in CS-MSCs are rescued by the targeted correction of mutant *ERCC6*. We next map the transcriptomic landscapes in CS-iPSCs and GC-iPSCs and their somatic stem cell derivatives (MSCs and NSCs) in the absence or presence of ultraviolet (UV) and replicative stresses, revealing that defects in DNA repair account for CS pathologies. Moreover, we generate autologous GC-MSCs free of pathogenic mutation under a cGMP (Current Good Manufacturing Practice)-compliant condition, which hold potential for use as improved biomaterials for future stem cell replacement therapy for CS. Collectively, our models demonstrate novel disease features and molecular mechanisms and lay a foundation for the development of novel therapeutic strategies to treat CS.

**Electronic supplementary material:**

The online version of this article (10.1007/s13238-019-0623-2) contains supplementary material, which is available to authorized users.

## INTRODUCTION

Cockayne syndrome (CS) is an autosomal recessive disorder characterized by progressive multisystem clinical features, including cachectic dwarfism, clinical photosensitivity, progressive neurological degeneration, and premature aging (Karikkineth et al., 2017). Two genes that are defective in Cockayne syndrome, *CSA*/*ERCC8* (ERCC excision repair 8, CSA ubiquitin ligase complex subunit) and *CSB*/ERCC6 (ERCC excision repair 6, chromatin remodeling factor), have been identified. To date, two-thirds of CS patients have been linked to mutations in the *CSB*/*ERCC6* gene, and one-third of CS patients have been linked to mutations in the *CSA*/*ERCC8* gene. At least 78 different mutations in *ERCC6*, including typical missense mutations, frameshifts, and deletions, have been identified (Cleaver et al., 2009; Laugel, 2013). However, the underlying molecular mechanisms linking genotype to phenotype need to be clarified.

DNA damage caused by exogenous ultraviolet (UV) radiation-induced photoproducts or similar chemically induced products is sensed by the cellular nucleotide excision repair (NER) system (Friedberg, 2001, 2003; Cleaver et al., 2009; McKay and Cabrita, 2013). The NER system consists of two pathways: global genomic repair (GGR), in which damage to DNA regions not undergoing transcription is repaired, and transcription-coupled repair (TCR), in which damage to transcribed DNA regions is repaired. Bulky DNA adducts usually block transcription elongation by RNA polymerase II (RNAPII); then, the arrested RNAPII initiates the repair of transcription-blocking DNA lesions by TCR to permit the efficient recovery of mRNA synthesis. If TCR cannot be executed, widespread sustained transcription blockage eventually leads to apoptosis (McKay and Cabrita, 2013). ERCC6 is an ATP-stimulated ATPase that is required for the ubiquitylation of the carboxyterminal domain of RNAPII in TCR and the recovery of mRNA synthesis. In addition, ERCC6 has been reported as a member of the SWI/SNF family of proteins that contain a nucleotide-binding site and play a role in chromatin maintenance and remodelling by modulating the negative supercoiling of DNA and facilitating DNA strand exchange, possibly through the recruitment of the histone acetyltransferase p300 (Newman et al., 2006; Cleaver et al., 2009; Velez-Cruz and Egly, 2013).

Mice deficient for *Ercc6* or *Ercc8* have been generated and used to mimic mild CS symptoms, including fat tissue reduction, photoreceptor cell loss, and mild but characteristic nervous system pathology (van der Horst et al., 1997, 2002; Gorgels et al., 2007; Jaarsma et al., 2011). These mild CS mouse models are converted to severe CS models with short life spans, progressive nervous system degeneration and cachectic dwarfism after synergistic complete inactivation of global genome NER. For example, previous studies have demonstrated the simultaneous deleterious effects of intercrossing xeroderma pigmentosum (XP) (*Xpa*^−/−^ or *Xpc*^−/−^) mice with CS (*Csa*^−/−^, *Csb*^−/−^, *Xpd*^*XPCS*^) mice, which results in double mutants with very short life spans and dramatic progeroid features (Murai et al., 2001; Andressoo et al., 2006; van der Pluijm et al., 2007). Due to the differences in genetic and anatomic features between humans and mice, a human CS model needs to be established to reveal the cellular defects and molecular mechanisms for translation into a CS treatment.

**Figure 1:**
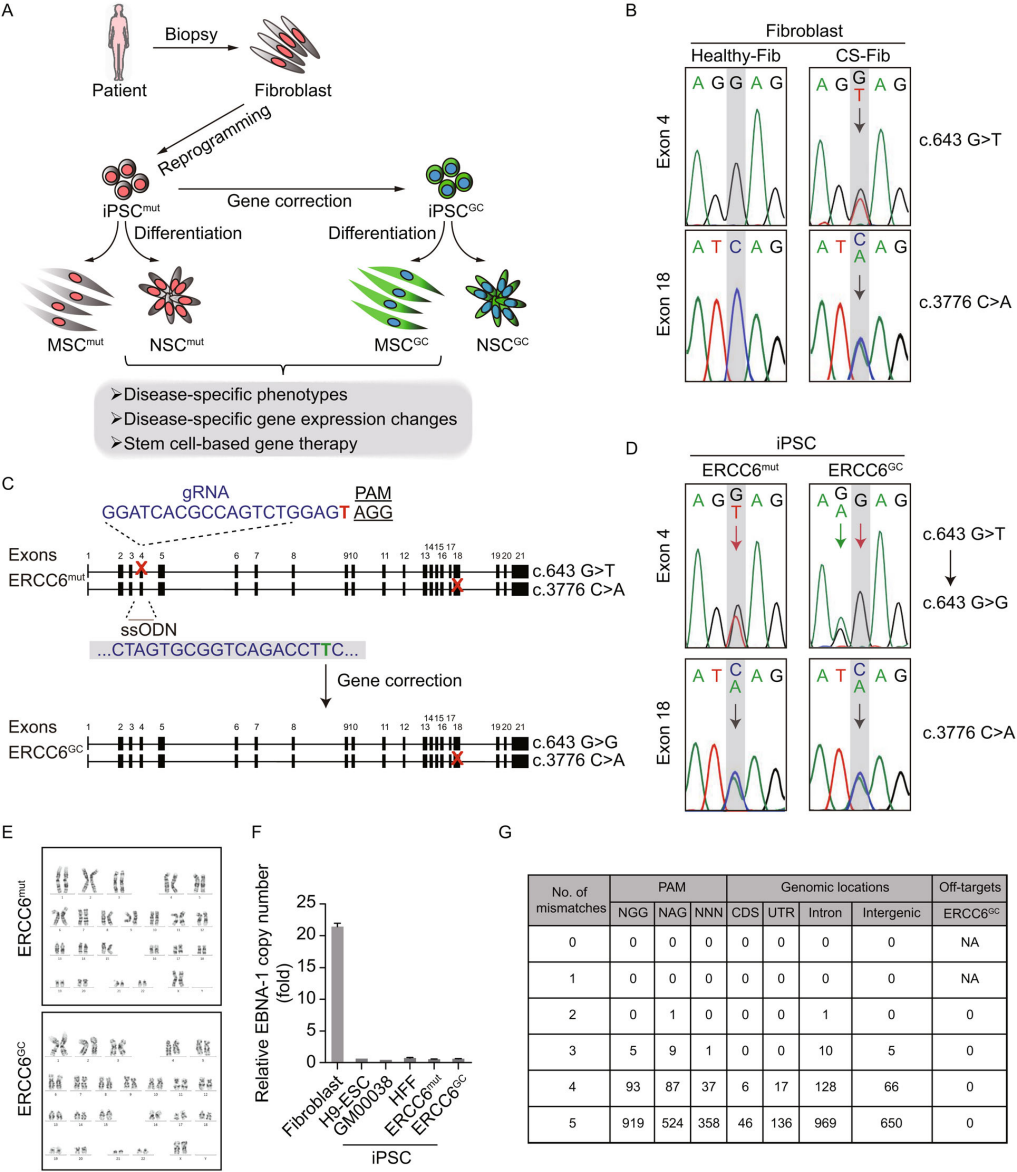
**Generation of CS-iPSCs and gene-corrected CS-iPSCs**. (A) Schematic diagram of the generation of CS-iPSCs and GC-iPSCs, as well as their adult stem cell derivatives, for modelling Cockayne syndrome. “Mut” represents mutant, “GC” represents gene corrected. (B) Genotype validation of two heterozygous mutations in the *ERCC6* gene by genomic DNA sequencing. Fibroblasts isolated from a healthy individual were used as a control. (C) Strategy for correcting the *ERCC6*^+/G643T^ mutation by the CRISPR/Cas9 system. The sequence of the gRNA is shown with the PAM sequence. Red crosses represent mutations in exon 4 and exon 18. The single-stranded oligodeoxynucleotide (ssODN) carrying a silent mutation (marked in green) was used as a repair template. (D) The correction of the *ERCC6*^+/G643T^ mutation was verified by genomic DNA sequencing. The red arrow highlights the corrected base pair. The green arrow indicates the inclusion of silent mutation introduced by the exogenous ssODN template. ERCC6^mut^ represents CS-iPSCs, ERCC6^GC^ represents GC-iPSCs. (E) Karyotyping analysis of CS-iPSCs and GC-iPSCs indicating their normal karyotypes. (F) No residual episomal vector element EBNA-1 was observed in CS-iPSCs or GC-iPSCs by qPCR analysis. CS-fibroblasts were electroporated with pCXLE-hOCT3/4-shp53-F, pCXLE-hSK and pCXLE-hUL. The fibroblasts were cultured for 4 more days after electroporation and then collected as the positive control, and human ESCs (line H9), GM00038-iPSCs and HFF-iPSCs were used as negative controls. Data are shown as the mean ± SEM, *n* = 3. (G) No off-target mutations were observed in GC-iPSCs. Whole-genome sequencing was applied to detect potential off-target mutations in the GC-iPSC sample. NA, not applicable

In this study, we report the generation of induced pluripotent stem cells (iPSCs) from the fibroblasts of a CS patient bearing two novel heterogeneous mutations in the *ERCC6* gene: c.643G>T in exon 4 and c.3776C>A in exon 18. We further derived gene-corrected CS-iPSCs (GC-iPSCs) using the CRISPR/Cas9-mediated gene editing technique. CS-iPSCs and GC-iPSCs were further differentiated into mesenchymal stem cells (MSCs) and neural stem cells (NSCs). Gene correction resulted in the effective restoration of DNA repair abilities and the alleviation of apoptosis and premature senescence, especially after exposure to UV irradiation or replicative stress (Fig. 1A). RNA sequencing analysis indicated that the compromised DNA repair and cell cycle deregulation observed in CS cells account for various CS cellular pathologies. Finally, we obtained gene-corrected CS-iPSC-derived MSCs under a cGMP (Current Good Manufacturing Practice)-compliant condition, which display promising potential in autologous stem cell therapy.

## RESULTS

### Generation of non-integrative iPSCs from a CS patient

**Figure 2:**
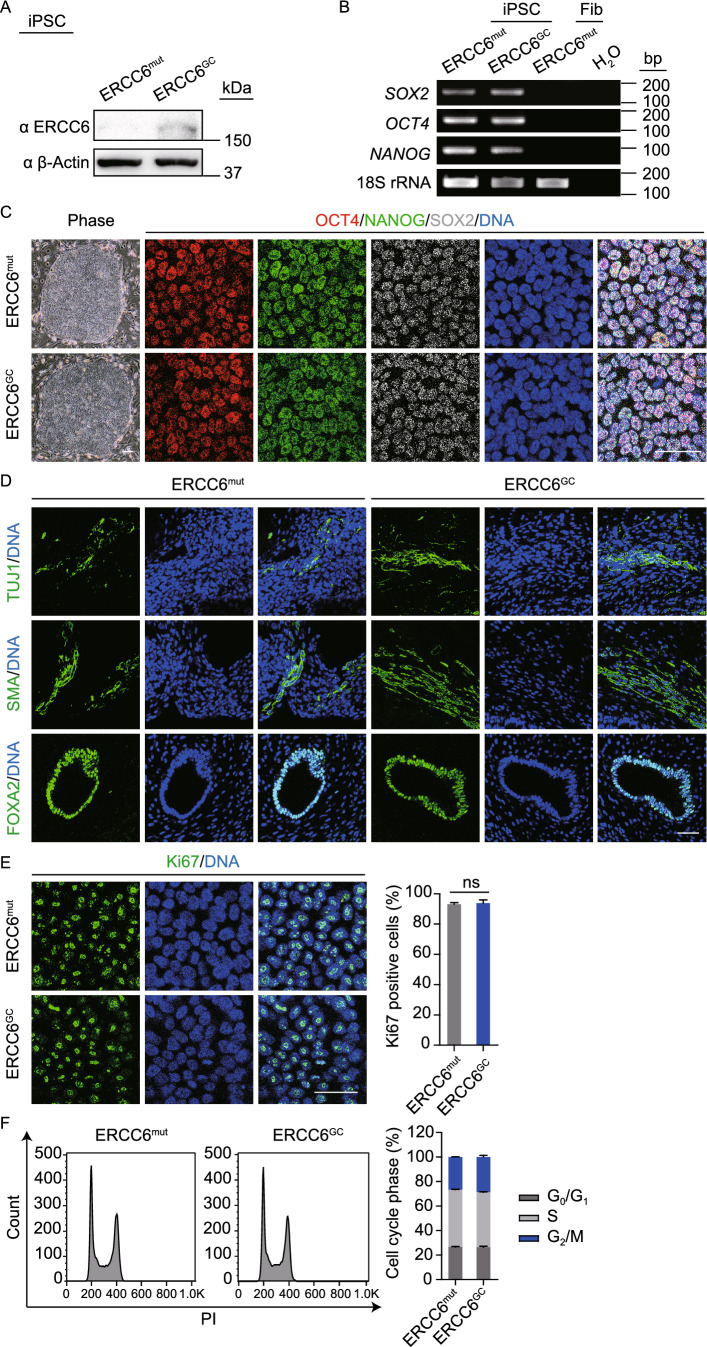
**Characterization of CS-iPSCs and gene-corrected CS-iPSCs**. (A) Western blot analysis showing increased protein levels of ERCC6 in GC-iPSCs. β-Actin was used as the loading control. (B) RT-PCR analysis of the pluripotency markers *SOX2*, *OCT4*, and *NANOG* in the CS-iPSCs and GC-iPSCs. 18S rRNA was used as the loading control. (C) Immunostaining of CS-iPSCs and GC-iPSCs for the pluripotency markers OCT4, NANOG, and SOX2. Nuclei were stained with Hoechst 33342. Scale bar, 50 μm. (D) Immunostaining of TUJ1 (ectoderm), SMA (mesoderm), and FOXA2 (endoderm) in teratomas derived from CS-iPSCs and GC-iPSCs. Nuclei were stained with Hoechst 33342. Scale bar, 50 μm. (E) The percentages of Ki67-positive cells in CS-iPSCs and GC-iPSCs were determined and compared. Nuclei were stained with Hoechst 33342. Scale bar, 50 μm. Data are presented as the mean ± SEM, *n* = 3, ns, not significant. (F) Cell cycle profiles showing comparable percentages of different cell cycle phases in CS-iPSCs and GC-iPSCs by PI staining. Data are presented as the mean ± SEM, *n* = 3

We first isolated human primary fibroblasts from a Chinese CS patient and verified the presence of two nonsense mutations, c.643G>T (p.E215X) in exon 4 and c.3776C>A (p.S1259X) in exon 18, located at different alleles of the *ERCC6* gene by genomic DNA sequencing analysis (Fig. 1B). To generate patient-specific iPSCs (CS-iPSCs), a cocktail of integration-free episomal vectors expressing the reprogramming factors OCT4, SOX2, KLF4, L-MYC, LIN28, and sh-p53 was electroporated into fibroblasts according to a modified reprogramming protocol, as previously described (Hishiya and Watanabe, 2004; Okita et al., 2011; Liu et al., 2014; Ding et al., 2015; Fu et al., 2016; Wang et al., 2017; Ling et al., 2019). The derived iPSCs displayed normal karyotypes, and no residual episomal reprogramming vector element was detected in established CS-iPSCs (Fig. 1E and 2F). In addition, CS-iPSCs expressed comparable levels of pluripotency markers, including NANOG, OCT4, and SOX2 (Fig. 2B and 2C). After being implanted subcutaneously into immunocompromised mice, CS-iPSCs were able to form teratomas comprising cells from three germ lineages, as indicated by TUJ1, SMA and FOXA2 expression (Fig. 2D). These observations indicated that iPSCs bearing the CS-specific *ERCC6* mutation display normal pluripotency.

### Targeted gene correction of the *ERCC6* mutation by CRISPR/Cas9 system

To better elucidate the pathogenic mechanism underlying CS, we generated isogenic gene-corrected iPSC lines by targeted gene editing of one of the two compound heterozygous *ERCC6* mutations. Using the CRISPR/Cas9 system, we electroporated an expression vector encoding mCherry and a guide RNA targeting the mutation in exon 4, a plasmid for Cas9-2A-GFP, and the single-stranded oligodeoxynucleotide (ssODN) template into CS-iPSCs (Wang et al., 2017). After fluorescence-activated cell sorting (FACS) for mCherry (guide RNA) and GFP (Cas9) double-positive cells, gene-corrected CS-iPSC clones were successfully obtained (Fig. 1C). Site-specific gene correction of the c.643G>T mutation was confirmed by genomic DNA sequencing (Fig. 1D). As the exogenous repair template ssODN was designed to contain a silent mutation, the introduced silent mutation was also found in the GC-iPSC clones, further confirming successful gene editing at the corresponding genomic target sites (Fig. 1D). Similar to CS-iPSCs, we did not detect any residual episomal reprogramming vectors in GC-iPSCs (Fig. 1F). Whole-genome DNA sequencing indicated no mutations in potential off-target sites after gene editing (Fig. 1G). GC-iPSCs also showed a normal karyotype (Fig. 1E). Western blots demonstrated elevated levels of the ERCC6 protein in GC-iPSCs (Fig. 2A), implying that the correction of the pathogenic mutation recovered the protein expression of ERCC6. Additionally, GC-iPSCs normally expressed pluripotency markers, including OCT4, NANOG, and SOX2 (Fig. 2B and 2C), and formed teratomas *in vivo* (Fig. 2D). CS-iPSCs and GC-iPSCs were cultured for more than 50 passages without showing abnormal growth kinetics (Fig. 2E and 2F). Unlike the previous study (Andrade et al., 2012), we did not observe elevated cellular reactive oxygen species (ROS) in CS-iPSCs compared to GC-iPSCs (Fig. S3A). In addition, RT-qPCR demonstrated that the expression levels of genes involved in the oxidative stress response were comparable between GC-iPSCs and CS-iPSCs (Fig. S3B). Taken together, these results indicated that we successfully generated GC-iPSCs exhibiting normal pluripotency.

### Alleviation of aging defects in gene-corrected CS-MSCs

**Figure 3:**
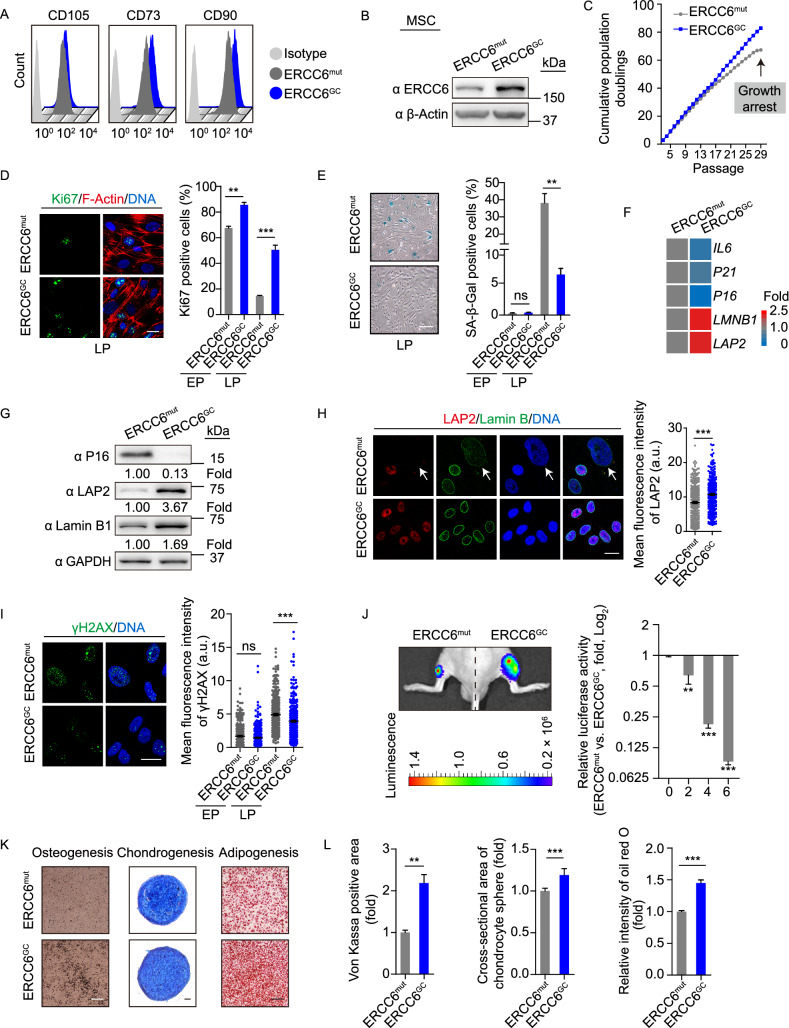
**Alleviated cellular senescence in gene-corrected CS-MSCs**. (A) FACS analysis indicating the expression of the cell surface markers CD73, CD90 and CD105 in CS-MSCs and GC-MSCs. ERCC6^mut^ represents CS-MSCs, ERCC6^GC^ represents GC-MSCs. (B) Western blot analysis showing increased protein levels of ERCC6 in GC-MSCs. β-Actin was used as the loading control. (C) Growth curves showing the cumulative population doublings of CS-MSCs and GC-MSCs. (D) Immunostaining of Ki67 showing the decreased cell proliferation of CS-MSCs compared to GC-MSCs. The percentages of Ki67-positive cells are shown in the right panel. Scale bar, 20 μm. Data are presented as the mean ± SEM, *n* = 3, **P < 0.01, ***P < 0.001. EP, early passage (P6); LP, late passage (P28). (E) SA-β-Gal staining of CS-MSCs and GC-MSCs at EP (P6) and LP (P28), respectively. The percentages of SA-β-Gal-positive cells are shown in the right panel. Scale bar, 50 μm. Data are presented as the mean ± SEM, *n* = 3, **P < 0.01, ns, not significant. (F) RT-qPCR analysis of the expression of senescence markers in CS-MSCs and GC-MSCs at passage 28. The mRNA levels were normalized to CS-MSCs. (G) Western blot analysis of P16, LAP2 and Lamin B1 in CS-MSCs and GC-MSCs. GAPDH was used as the loading control. (H) Immunostaining of LAP2 and Lamin B in CS-MSCs and GC-MSCs. The relative intensity of LAP2 was measured with ImageJ software, and the data are shown as the mean ± SEM, ***P < 0.001. More than 300 nuclei for each group were used for calculations. Scale bar, 20 μm. a.u., arbitrary units. (I) Immunostaining of γH2AX in CS-MSCs and GC-MSCs. The relative intensity of γH2AX was measured with ImageJ software, and the data are shown as the mean ± SEM, ***P < 0.001. More than 300 nuclei for each group were used for calculations. Scale bar, 20 μm. a.u., arbitrary units. (J) Accelerated attrition of CS-MSCs *in vivo* was detected by an *in vivo* imaging system (IVIS). CS-MSCs (1 × 10^6^, left) and GC-MSCs (1 × 10^6^, right) (passage 25) infected with luciferase lentivirus were injected into the tibialis anterior (TA) muscles of immunodeficient mice. Luciferase activities were imaged and quantified at days 0, 2, 4, and 6 after transplantation. Data are presented as the ratios of the luciferase intensity of CS-MSCs to that of GC-MSCs (fold), mean ± SD, *n* = 3, **P < 0.01, ***P < 0.001. (K) Comparative analysis of the osteogenic, chondrogenic and adipogenic differentiation potential of CS-MSCs and GC-MSCs. Von Kossa, Alcian blue, and oil red O staining were used to characterize osteoblasts, chondrocytes, and adipocytes, respectively. Scale bar, 50 μm. (L) The intensity of von Kossa staining was calculated by ImageJ and compared in the left panel. Data are presented as the mean ± SEM, *n* = 3, **P < 0.01. The cross-sectional area of chondrocyte spheres was measured and is shown in the middle panel. Data are presented as the mean ± SD, *n* = 14, ***P < 0.001. The relative intensity of oil red O was measured and is shown in the right panel. Data are presented as the mean ± SEM, *n* = 3, ***P < 0.001

CS patients frequently exhibit musculoskeletal abnormalities, such as kyphosis, contracture and osteoporosis (Hishiya and Watanabe, 2004; Karikkineth et al., 2017). MSCs are multipotent mesodermal cells that can differentiate into a variety of mesodermal cell types, including osteoblasts, chondrocytes, and adipocytes, which serve as a good cell model for investigating the accelerated degeneration of mesodermal tissues caused by genetic mutations (Liu et al., 2014; Zhang et al., 2015, 2019; Kubben et al., 2016; Li et al., 2016; Pan et al., 2016; Geng et al., 2018; Wang et al., 2018b; Wu et al., 2018; Yan et al., 2019). Therefore, we first differentiated CS-iPSCs and GC-iPSCs into MSCs to investigate whether *ERCC6* mutations could result in accelerated attrition of the MSC pool. Both CS-MSCs and GC-MSCs were positive for mesenchymal progenitor markers, including CD73, CD90 and CD105 (Fig. 3A). Consistent with the successful correction of *ERCC6* gene mutation, increased ERCC6 protein content was observed in GC-MSCs (Fig. 3B). Next, we investigated whether normal ERCC6 activity is required for maintaining the cellular homeostasis of MSCs. Compared to isogenic gene-corrected control cells, CS-MSCs displayed features characteristic of premature senescence under replicative stress, including the early onset of cell growth arrest, reduced Ki67-positive cells, and increased senescence-associated (SA)-β-Gal activity (Fig. 3C–E). In addition, the expression levels of senescence markers, including P16, P21 and IL-8, were upregulated, while the geroprotective proteins Lamin B1 and LAP2 were downregulated in CS-MSCs relative to GC-MSCs at late passages (Fig. 3F–H). In line with the essential role of ERCC6 in NER, CS-MSCs exhibited increased expression of the DNA damage marker γH2AX (Fig. 3I), indicating compromised DNA repair in ERCC6-deficient MSCs. Next, we investigated whether CS-MSCs underwent accelerated attrition *in vivo.* Implanting CS-MSCs and GC-MSCs expressing luciferase into the tibialis anterior (TA) muscle of immunodeficient mice resulted in accelerated *in vivo* decay in CS-MSCs compared to GC-MSCs (Fig. 3J). Furthermore, we compared the multipotent differentiation potential of CS-MSCs and GC-MSCs. Relative to GC-MSCs, CS-MSCs exhibited impaired differentiation abilities towards osteoblasts, chondrocytes and white adipocytes (Fig. 3K and 3L). Altogether, these results showed that CS-MSCs displayed typical premature cellular senescence, which was rescued by the targeted correction of mutant *ERCC6*.

### Gene-corrected CS-MSCs display recovered DNA repair ability and resistance to UV-induced apoptosis and cell cycle arrest

**Figure 4:**
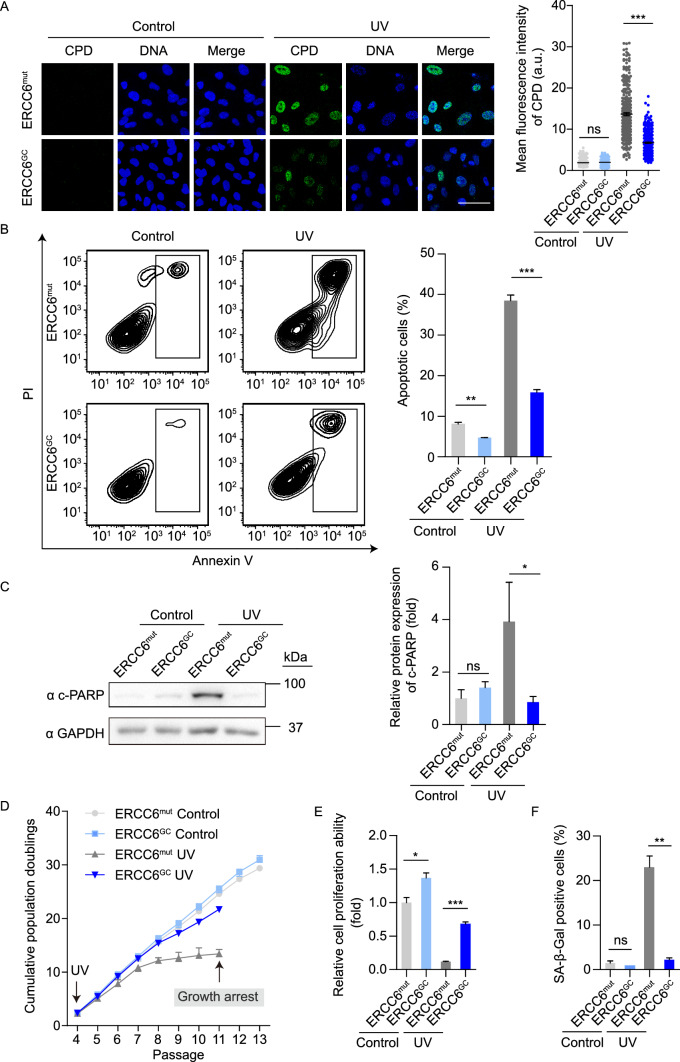
**Gene-corrected CS-MSCs display recovered DNA repair ability and counteract UV-induced apoptosis and senescence**. (A) CPD immunostaining in CS-MSCs and GC-MSCs in the absence or presence of 10 J/m^2^ UV exposure. Nuclei were stained with Hoechst 33342. Scale bar, 50 μm. More than 300 nuclei for each group were used for calculation. The data are shown as the mean ± SEM, ns, not significant, ***P < 0.001. a.u., arbitrary units. (B) Apoptosis analysis of CS-MSCs and GC-MSCs at 48 h after 10 J/m^2^ UV irradiation. Quantitative data are presented as the mean ± SEM, *n* = 3, **P < 0.01, ***P < 0.001. (C) Western blots showing PARP cleavage in CS-MSCs and GC-MSCs in the absence or presence of 10 J/m^2^ UV exposure. GAPDH was used as a loading control. Quantitative data are presented as the mean ± SD, *n* = 3, ns, not significant, *P < 0.05. (D) Growth curves showing the cumulative population doublings of CS-MSCs and GC-MSCs in the absence (control) or presence (UV) of 1 J/m^2^ UV exposure at each passage starting from passage 4. (E) Clonal expansion assay showing the cell proliferation ability of CS-MSCs and GC-MSCs in the absence (control) or presence (UV) of 1 J/m^2^ UV exposure at passage 10. The cells were stained with crystal violet after two weeks of culture, and the relative intensity of the crystal violet staining was quantified. Data are presented as the mean ± SEM, *n* = 3, *P < 0.05, ***P < 0.001. (F) SA-β-Gal staining of CS-MSCs and GC-MSCs in the absence (control) or presence (UV) of 1 J/m^2^ UV exposure at passage 10. The percentages of SA-β-Gal-positive cells are shown in the right panel. Data are presented as the mean ± SEM, *n* = 3, **P < 0.01, ns, not significant

Next, we investigated whether mutations in *ERCC6* genes lead to impaired DNA damage repair ability after UV irradiation in MSCs. UV radiation usually results in the covalent dimerization of adjacent pyrimidines, typically thymine residues (thymine dimers), including cyclobutane pyrimidine dimers (CPDs) and (6-4) photoproducts (6-4PPs), in DNA (Setlow and Setlow, 1962; Friedberg, 2003; Cadet et al., 2005). Accordingly, we treated CS-MSCs and GC-MSCs with 10 J/m^2^ UV irradiation and examined the levels of intranuclear CPDs by immunostaining. Both CS-MSCs and GC-MSCs showed low levels of CPDs in the absence of UV irradiation; however, CS-MSCs exhibited more CPD-positive cells than GC-MSCs did at 48 h after UV irradiation (Fig. 4A). These results demonstrated that CS-MSCs were deficient in eliminating CPD photolesions after UV-induced DNA damage, and this ability was restored by *ERCC6* correction. We then explored whether CS-MSCs are hypersensitive to UV-induced cellular apoptosis. CS-MSCs and GC-MSCs were cultured in the presence or absence of 10 J/m^2^ UV irradiation. UV irradiation induced marked cellular apoptosis in CS-MSCs relative to GC-MSCs at 48 h after UV irradiation (Fig. 4B). Western blot analysis showed increased levels of cleaved PARP (c-PARP) in CS-MSCs following UV treatment (Fig. 4C). In addition, we treated MSCs with a lower dose (1 J/m^2^) of UV light at each passage starting from passage 4. In this context, relative to GC-MSCs, CS-MSCs displayed compromised self-renewal ability and increased SA-β-Gal-positive cells (Fig. 4D–F), indicating that the ERCC6 deficiency rendered MSCs sensitive to replicative stress under low-dose chronic UV irradiation. Thus, CS-specific MSCs exhibited impaired DNA repair ability and increased susceptibility to UV-induced injury, and these phenotypes were rescued by the genetic correction of the pathogenic mutation.

### Gene-corrected CS-NSCs display improved NER ability and reduced susceptibility to UV-induced apoptosis

**Figure 5:**
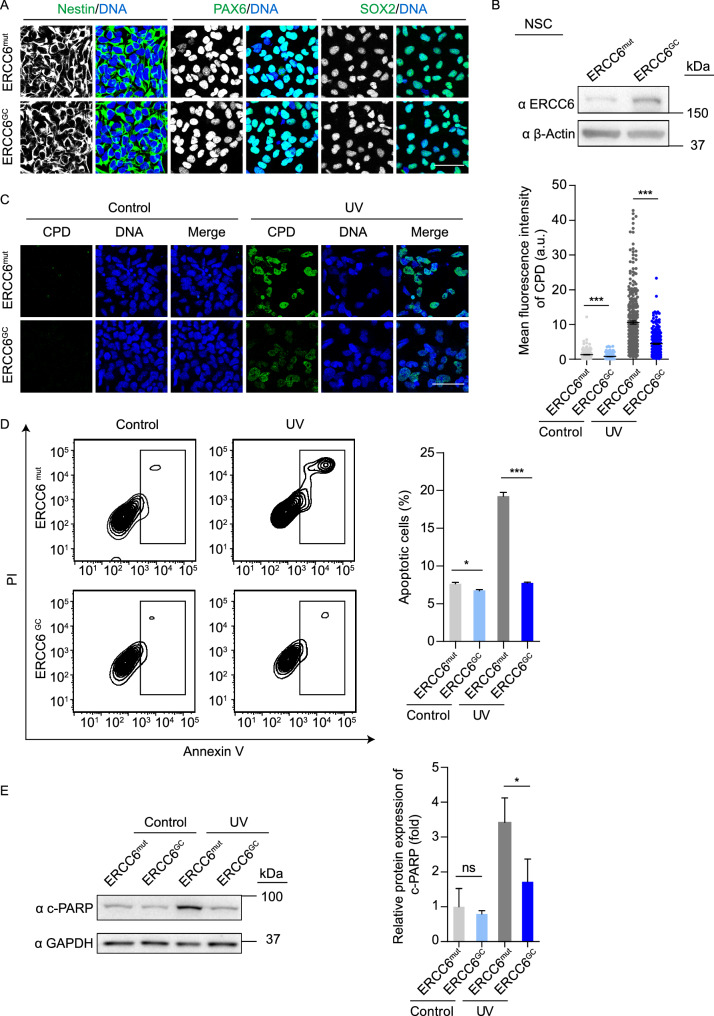
**Gene-corrected CS-NSCs show increased NER ability and decreased susceptibility to UV-induced apoptosis**. (A) Immunostaining of the NSC markers Nestin, PAX6, and SOX2 in the CS-NSCs and GC-NSCs. The nuclei were stained with Hoechst 33342. Scale bar, 50 μm. ERCC6^mut^ represents CS-NSCs, ERCC6^GC^ represents GC-NSCs. (B) Western blot analysis showing increased protein levels of ERCC6 in GC-NSCs. β-Actin was used as the loading control. (C) CPD immunostaining in CS-NSCs and GC-NSCs in the absence or presence of 5 J/m^2^ UV exposure. Nuclei were stained with Hoechst 33342. Scale bar, 50 μm. Over 300 nuclei were used for calculations. The data are shown as the mean ± SEM, ***P < 0.001. a.u., arbitrary units. (D) Apoptosis analysis of CS-NSCs and GC-NSCs at 48 h after 5 J/m^2^ UV irradiation. Quantitative data are presented as the mean ± SEM, *n* = 3, *P < 0.05, ***P < 0.001. (E) Western blots showing PARP cleavage in CS-NSCs and GC-NSCs in the absence or presence of 5 J/m^2^ UV exposure. GAPDH was used as a loading control. Quantitative data are presented as the mean ± SD, *n* = 3, *P < 0.05, ns, not significant

Due to the presence of obvious symptoms of neurodegeneration in CS patients (Cleaver et al., 2009; Natale, 2011; Laugel, 2013; Shehata et al., 2014), we next differentiated CS-iPSCs and GC-iPSCs into NSCs (referred to as CS-NSCs and GC-NSCs, respectively). Both CS-NSCs and GC-NSCs showed typical neural progenitor morphology and expressed the NSC markers Nestin, PAX6 and SOX2 (Fig. 5A). Western blots confirmed the increased protein expression of ERCC6 in GC-NSCs compared to that in uncorrected CS-NSCs (Fig. 5B). To investigate whether mutations in the *ERCC6* gene impair the DNA repair ability of NSCs, we treated CS-NSCs and GC-NSCs with 5 J/m^2^ UV irradiation and then examined the levels of intranuclear CPDs. Similar to the results obtained with MSCs, higher levels of CPDs were observed in CS-NSCs than in GC-NSCs at 48 h after UV irradiation, indicating that targeted gene correction effectively rescued the hypersensitivity of CS-NSCs to UV irradiation (Fig. 5C). Consistent with this finding, gene correction resulted in decreased cellular apoptosis in CS-NSCs in the presence of UV treatment (Fig. 5D and 5E). Altogether, these results indicated that CS-NSCs, which are characterized by a DNA repair deficit, were prone to UV-induced apoptosis, while genetic correction resulted in the restoration of these phenotypic defects.

### The *ERCC6* mutation results in gene expression changes associated with impaired DNA damage repair, chromatin disorganization, and compromised cell proliferation

**Figure 6:**
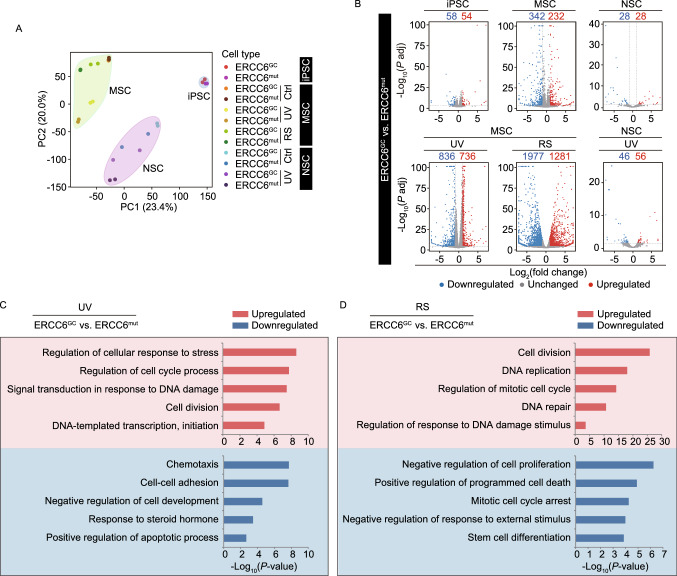
**The global gene expression profiles of CS-iPSCs and gene-corrected CS-iPSCs and their adult stem cell derivatives**. (A) PCA of CS cells and GC cells in the absence or presence of UV (Ctrl or UV), as well as under replicative senescence (RS) stress. Each point represents a sample. Data points were computed based on Log_2_(FPKM + 1). (B) Volcano plots showing the differentially expressed genes between CS-iPSCs and GC-iPSCs, between CS-MSCs and GC-MSCs, and between CS-NSCs and GC-NSCs in the absence of UV (the upper panel) or in the presence of UV (the lower panel, UV), or under RS stress (the lower panel, RS). Red represents upregulated genes, and blue represents downregulated genes. (C) Gene Ontology Biological Process (GO-BP) enrichment analysis of significantly upregulated/downregulated genes in GC-MSCs compared to CS-MSCs upon UV treatment. Red represents upregulated genes, and blue represents downregulated genes. (D) Gene Ontology Biological Process (GO-BP) enrichment analysis of significantly upregulated/downregulated genes in GC-MSCs compared to CS-MSCs under RS stress. Red represents upregulated genes, and blue represents downregulated genes

To investigate whether gene expression profiles were disrupted in CS-specific iPSCs, MSCs and NSCs, we performed genome-wide RNA sequencing (RNA-seq) analysis (Figs. 6, S1 and S2). Principal component analysis (PCA) showed that the RNA profiles of MSCs, iPSCs and NSCs were separated as three independent subgroups (Fig. 6A), implying the existence of unique RNA expression patterns in each cell type. While there were minimal gene expression changes between CS-iPSCs and GC-iPSCs and between CS-NSCs and GC-NSCs, the mutation of *ERCC6* resulted in marked changes in the transcriptome of MSCs (Figs. 6B and S1C). These observations were in line with the most striking phenotypes in CS-MSCs relative to their gene-corrected counterparts under basal culture conditions (Fig. 3C–E). UV treatment results in an increased difference in transcriptional profiles between GC-MSCs and CS-MSCs and between GC-NSCs and CS-NSCs (Figs. 6B and S1C). Notably, UV treatment induced dramatic gene expression changes in CS-specific MSCs and CS-specific NSCs (Fig. S1E), which were associated with increased DNA damage, impaired transcription, and compromised cell growth; these changes, however, became insensitive in *ERCC6*-corrected MSCs and NSCs, indicating that gene correction resulted in the restoration of normal transcriptional and DNA repair activity under DNA damage stress (Fig. 6C). After extensive passaging, we also observed a panel of upregulated genes related to cell division and DNA damage repair in *ERCC*6-corrected MSCs compared to diseased MSCs (Fig. 6D), which is in line with the rescue of premature cellular senescence in gene-corrected MSCs (Fig. 3C–J). Collectively, these transcriptomic changes support the improved cell proliferation and increased DNA damage repair ability in *ERCC6*-corrected adult stem cells.

### Gene-corrected CS-MSCs produced in accordance with cGMP compliance guidelines show alleviated senescence and increased resistance to UV-induced apoptosis

**Figure 7:**
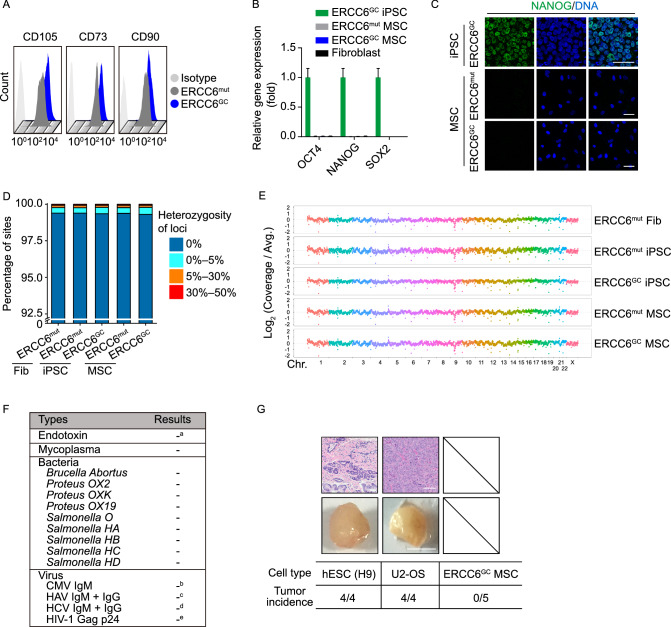
**Safety analysis of gene-corrected CS-MSCs obtained under a cGMP-compliant condition**. (A) FACS analysis indicated the expression of the cell surface markers CD73, CD90 and CD105 in CS-MSCs and GC-MSCs. (B) RT-qPCR analysis of the expression of pluripotency markers *OCT4*, *NANOG*, and *SOX2* in CS-MSCs and GC-MSCs. GC-iPSCs and CS-fibroblasts were used as positive and negative controls, respectively. Data are presented as the mean ± SEM, *n* = 3. (C) Immunostaining of the pluripotency marker NANOG in CS-MSCs and GC-MSCs. GC-iPSCs were used as a positive control, Scale bar, 50 μm. (D) Whole-genome sequencing of single-nucleotide variants (SNVs) in CS-fibroblasts, CS-iPSCs, GC-iPSCs, CS-MSCs and GC-MSCs. Sites with a heterozygosity percentage ranging between 0% and 30% were considered as SNV sites, and sites with a heterozygosity of >30% were considered as single-nucleotide polymorphisms (SNPs). (E) Whole-genome sequencing of copy number variations (CNVs) in CS-fibroblasts, CS-iPSCs, GC-iPSCs, CS-MSCs and GC-MSCs. Each point represents normalized coverage depth of each 500-kb genomic region of each chromosome. (F) Sterility and pathogen testing of the conditioned medium of GC-MSCs. ^a^ Endotoxin was identified as negative when the concentration was < 0.25 EU/mL. ^b^ CMV was identified as negative when the ratio of the OD_450_ value of sample to the cut-off value (S/Co) was < 1.0. ^c^ HAV was identified as negative when the ratio of the cut-off value to the OD450 nm value of the sample (Co/S) was < 0.9. ^d^ HCV was identified as negative when the ratio of the OD_450_ value of the sample to the cut-off value (S/Co) was < 0.9. ^e^ HIV-1 was identified as negative when the concentration = 0 pg/mL. (G) Evaluation of the potential tumorigenesis risk of GC-MSCs *in vivo*. A subcutaneous injection of GC-MSCs was performed in immune-deficient mice. Human ESC (line H9) and U2-OS osteosarcoma cell lines were also implanted independently as positive controls. Representative images in the lower panel showing the teratoma and tumor formed from positive cells two months after transplantation, Scale bar, 0.5 cm. HE staining of a teratoma and tumor were shown in the upper panel. Scale bar, 100 μm. The *in vivo* tumor-formation incidence of each cell type was calculated. *n* = 4 for each positive cell group, *n* = 5 for the GC-MSC group

Human mesenchymal stem cells hold the potential to be used for the treatment of aging-related disorders (Orozco et al., 2011, 2013, 2014; Golpanian et al., 2016, 2017; Tompkins et al., 2017; Yang et al., 2017; Yan et al., 2019). We next tested whether ERCC6-corrected CS-MSCs can be produced under a cGMP-compliant condition. Accordingly, we derived MSCs from iPSCs using a serum-free, animal component-free differentiation medium. The differentiation protocol was slightly modified from the serum-containing procedure (see experimental method). FACS analysis demonstrated that the derived MSCs expressed the mesenchymal progenitor cell-specific markers CD73, CD90 and CD105 (Fig. 7A). The absence of pluripotent stem cell contamination in the derived MSCs was verified by RT-qPCR and immunostaining assays (Fig. 7B and 7C). Whole-genome DNA sequencing further validated the genomic integrity during somatic cell reprogramming, gene correction, and directed differentiation to MSCs (Fig. 7D and 7E). Sterility and pathogen testing demonstrated that there was no endotoxin, mycoplasma, bacteria, or virus contamination in the culture medium of the GC-MSCs (Fig. 7F). To evaluate any potential risk of tumorigenesis *in vivo*, immunodeficient mice were subcutaneously injected with the *ERCC6*-corrected MSCs. Human ESC (line H9) and U2-OS osteosarcoma cell lines were implanted independently as positive controls. We observed that the GC-MSCs failed to form tumors, even at 8 months after implantation, in contrast with the teratomas formed from hESCs and tumors formed from U2-OS cells at 2 months post-injection (Fig. 7G).

**Figure 8:**
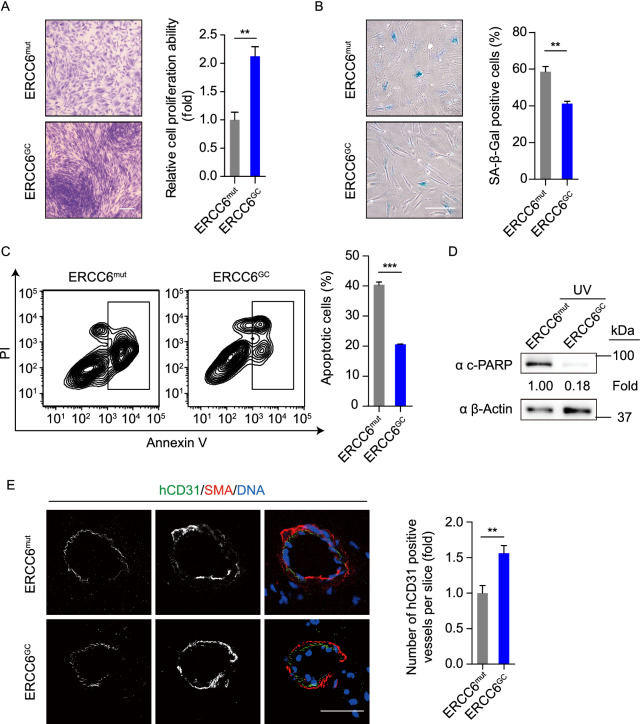
**Gene-corrected CS-MSCs generated under a cGMP-compliant condition displayed alleviated aging defects and decreased susceptibility to UV-induced apoptosis**. (A) Clonal expansion assay showing the cell proliferation ability of CS-MSCs and GC-MSCs. The cells were stained with crystal violet after a two-week culture, and the relative intensity of the crystal violet was quantified. Data are presented as the mean ± SEM, *n* = 4, **P < 0.01. Scale bar, 50 μm. (B) SA-β-Gal staining of CS-MSCs and GC-MSCs. The percentages of SA-β-Gal-positive cells are shown in the right panel. Data are presented as the mean ± SEM, *n* = 3, **P < 0.01. Scale bar, 50 μm. (C) Apoptosis analysis of CS-MSCs and GC-MSCs 48 h after 10 J/m^2^ UV irradiation. Quantitative data are presented as the mean ± SEM, *n* = 3, ***P < 0.001. (D) Western blots showing PARP cleavage of CS-MSCs and GC-MSCs in the presence of 10 J/m^2^ UV exposure. β-Actin was used as a loading control. (E) Fat pad transplantation with CS-MSCs and GC-MSCs. Left: representative immunofluorescent images showing neovascularization; right: the number of hCD31-positive vessels calculated based on 24 slices from inconsecutive frozen sections. Data are presented as the mean ± SD, *n* = 3 for each group, **P < 0.01. Scale bar, 50 μm

Phenotypically, compared to diseased MSCs, gene-corrected MSCs generated following the cGMP compliance standard displayed increased cell proliferation and attenuated cellular senescence (Fig. 8A and 8B). In addition, the GC-MSCs were insensitive to UV-induced apoptosis (Fig. 8C and 8D). Consistent with an improved activity, these GC-MSCs exhibited better tri-lineage differentiation potential towards osteoblasts, chondrocytes and adipocytes (Fig. S3C–D). A fat pad implantation assay further demonstrated the superior *in vivo* neovascularization ability of GC-MSCs (Fig. 8E). Altogether, we successfully generated *ERCC6*-corrected MSCs with normal functional activity under a cGMP-compliant condition.

## DISCUSSION

Although several mouse models exhibiting the clinical symptoms of CS have been generated and have provided valuable insights into the disease mechanism, there are still many differences in clinical features between CS patients and mouse models. For instance, in contrast to human CS patients, who do not develop skin cancer, *ERCC6* mutant mice show increased susceptibility to skin cancer (van der Horst et al., 1997, 2002). Thus, CS mouse models do not fully mimic the pathophysiology of CS patients, and the knowledge learned from animal models may be poorly translated to the clinic. CS patient-specific iPSCs were initially obtained by reprogramming fibroblasts from CS patients using retroviral vectors, and these cells exhibited an elevated cell death rate and increased ROS production (Andrade et al., 2012). Our study, however, did not identify increased oxidative stress or altered levels of *TXNIP* (Fig. S3A and S3B). These differences may be attributed to the reprogramming vectors. Luciana et al. used retroviral vectors, which may result in random genomic integration and genomic instability during the reprograming process. In addition, the same research group recently reported that CS-iPSC-derived neurons display reduced synapse density and altered neural network synchrony (Vessoni et al., 2016). Again, this study was based on a retroviral vector-mediated somatic reprograming technique. More importantly, due to the lack of an isogenic “disease-free” control iPSC line, it is hard to determine whether the phenotypic differences are caused by *ERCC6* gene mutations or genetic background variations between CS patients and control individuals. To faithfully recapitulate human CS pathogenesis, a reliable human iPSC-based disease model with isogenic gene-corrected cells is required. In this study, we generated transgene-free iPSCs from the fibroblasts of a CS patient bearing newly identified heterozygous disease-causing mutations in the *ERCC6* gene and obtained isogenic gene-corrected iPSCs using the CRISPR/Cas9 system. These iPSCs were further differentiated into two types of adult stem cells, MSCs and NSCs, which presented a panel of new disease phenotypes.

Although previous studies have reported that the deficiency of functional DNA repair proteins may hinder somatic cell reprogramming and teratoma formation *in vivo* (i.e., WRN (Shimamoto et al., 2014; Wang et al., 2018c), p53 (Kawamura et al., 2009), and Fanconi genes (Muller et al., 2012)), we did not observe any defects in the derivation or pluripotency of CS patient-specific iPSC lines. Moreover, *ERCC6* gene mutations did not compromise the chromosomal integrity of iPSCs, as indicated by karyotype analysis. Our study also provides proof of concept that CRISPR/Cas9-mediated gene editing may be amenable to correcting *ERCC6* mutation in a therapeutic context. Whole-genome DNA sequencing demonstrated minimal mutational load in patient iPSCs after targeted gene correction.

Although CS patients exhibit musculoskeletal abnormalities (Hishiya and Watanabe, 2004), there are limited reports concerning mesodermal cells. Using an iPSC-based system, we have for the first time generated CS-specific MSCs that display differentiation potential towards osteoblasts, chondrocytes and white adipocytes, and these cells serve as a good cell model to study mesodermal abnormalities in CS patients. Consistent with the premature degeneration of mesenchymal progenitor cells, CS-MSCs exhibit decreased cell proliferation, accelerated senescence, and compromised differentiation ability towards osteoblasts, chondrocytes and white adipocytes, which may constitute one of the causes of the observed defects in the musculoskeletal system. In addition, in agreement with previous reports showing confounding defects in the neural system in CS patients (Cleaver et al., 2009; Natale, 2011; Laugel, 2013; Sacco et al., 2013; Ciaffardini et al., 2014; Vessoni et al., 2016), our data indicated severe DNA repair defects and increased susceptibility to UV-induced apoptosis in CS-iPSC-derived NSCs, therefore providing in-depth mechanistic insights into CS-associated neurological disorders.

Regarding the molecular mechanism, we have generated the first *ERCC6* mutation-associated disease transcriptome landscapes of human MSCs and NSCs using an isogenic iPSC-based research system. Under normal culture conditions, mutation of *ERCC6* resulted in the most dramatic gene expression changes in MSCs relative to NSCs and iPSCs. Consistent with this finding, CS-specific MSCs demonstrated cell type-specific accelerated senescence after serial passaging. These results suggest that the attrition of the MSC pool and the resulting mesodermal defects are a major syndrome of CS. UV radiation generates photoproducts in genomic DNA that promote genetic mutations that contribute to skin carcinogenesis or cellular senescence (Amaro-Ortiz et al., 2014; Kemp et al., 2017). In this study, we found that *ERCC6* mutant MSCs and NSCs were highly susceptible to UV radiation. A defect in the initiation of transcription by RNAPII in UV-treated CS and XP/CS cells has been observed in previous studies (Rockx et al., 2000; Yamada et al., 2002; Proietti-De-Santis et al., 2006; Velez-Cruz et al., 2013). In line with these results, we observed that transcriptional blockage was rescued in gene-corrected CS-MSCs after UV irradiation. In addition, the presence of the *ERCC6* mutation is associated with defects in gene expression linked to “cellular response to DNA damage”, “cellular response to stress” and “cell division”, indicating that the defective DNA repair in CS-specific adult stem cells mediates UV-induced cell phenotypic abnormalities. In addition, the mutation of *ERCC6* also led to gene expression changes related to “regulation of chromatin organization” in both NSCs and MSCs. Therefore, the pathogenesis of CS may involve a complex interplay among defects in DNA damage repair, chromatin organization, and cell cycle control.

In the context of disease therapy, stem cell-based replacement therapy holds great promise toward restoring tissue homeostasis, e.g., for premature aging disorders (Golpanian et al., 2017; Tompkins et al., 2017). We and others have produced adult stem cells and other terminally differentiated cells from iPSCs derived from various human aging-related disorders, including Hutchinson-Gilford progeria syndrome (HGPS), Werner syndrome (WS), Fanconi anemia (FA), XP, amyotrophic lateral sclerosis (ALS), and Parkinson’s disease (PD) (Liu et al., 2011a, 2012, 2014; Zhang et al., 2015; Fu et al., 2016; Wang et al., 2017). Using targeted gene editing techniques, we have also edited/corrected pathogenic mutations in these patient-derived iPSCs (Liu et al., 2011b, 2012, 2014; Wang et al., 2017). MSCs can differentiate into osteoblasts, chondrocytes, myocytes and adipocytes. Previous studies have shown that MSCs ameliorate aging frailty in clinical trials (Golpanian et al., 2016, 2017; Tompkins et al., 2017). Recently, the generation of allogeneic or autologous MSCs from pluripotent stem cells has emerged as a promising new strategy for stem cell-based therapy (Yang et al., 2017; Castro-Vinuelas et al., 2018; Soontararak et al., 2018; Yan et al., 2019). In the present study, we have derived MSCs from gene-corrected CS-iPSCs under a cGMP-compliant condition. These MSCs demonstrated superior cellular activity compared to uncorrected diseased cells, retained high genomic stability, and did not form tumors *in vivo*. Therefore, clinical-grade GC-MSCs may represent important biomaterials for achieving autologous stem cell treatment for CS.

In summary, the isogenic CS stem cell models established in this study provide a valuable platform for studying CS pathogenesis, discovering innovative drugs, and the development of new cell replacement therapies. The transcriptomic profiles underlying disease phenotypes may be useful for discovering biomarkers for diagnosis and the development of new therapeutic approaches.

## MATERIALS AND METHODS

### Antibodies and reagents

The primary antibodies used were as follows (company, catalogue number): anti-ERCC6 (Abcam, ab96098), anti-NANOG (Abcam, ab21624), anti-SOX2 (Santa Cruz, sc-17320), anti-OCT4 (Santa Cruz, sc-5279), anti-SMA (Sigma, A5228), anti-TUJ1 (Sigma, T2200), anti-FOXA2 (Cell Signaling Technology, 8186S), anti-CD90-FITC (BD Bioscience, 555595), anti-CD73-PE (BD Bioscience, 550257), anti-CD105-APC (BD Bioscience, 17-1057-42), anti-IgG-FITC (BD Biosciences, 555748), anti-IgG-PE (BD Biosciences, 555749), anti-IgG-APC (BD Biosciences, 555751), anti-Lamin B (Santa Cruz, sc-6217), anti-LAP2 (BD Bioscience, 611000), anti-Ki67 (ZSGB-BIO, ZM0166), anti-P16 (BD Bioscience, 550834), anti-γ-H2AX (Millipore, 05-636), anti-Nestin (Millipore, MAB5326), anti-PAX6 (Covance, PRB-278P), anti-CPD (Cosmo Bio, TMD-2), anti-cleaved PARP (Cell Signaling Technology, 9541), anti-β-Actin (Santa Cruz, sc69879), anti-GAPDH (Santa Cruz, sc-25778), and anti-hCD31 (BD Bioscience, 555445).

### Generation and genotyping of CS-specific fibroblasts

CS-specific fibroblasts were generated from the skin biopsy of a CS patient carrying two heterozygous *ERCC6* mutations: c.643G>T in exon 4 and c.3776C>A in exon 18. Fibroblasts were cultured with high-glucose DMEM (HyClone) containing 10% fetal bovine serum (FBS, Gemcell), 1% penicillin/streptomycin (Gibco), and 0.1 mmol/L non-essential amino acids (Gibco). Genotyping of CS-specific fibroblasts was performed using a genomic DNA PCR assay with the primers listed in Table S1. Genomic DNA from the fibroblasts of healthy donor was used as a control, as previously described (Fu et al., 2016).

### iPSC generation and culture

CS patient-specific iPSCs were generated by the electroporation of fibroblasts with episomal vectors, including pCXLE-hSK, pCXLE-hOCT3/4-shp53-F and pCXLE-hUL, as previously described (Okita et al., 2011; Liu et al., 2012, 2014; Fu et al., 2016; Wang et al., 2017). The derived iPSC lines were cultured on mitomycin C-treated MEF feeder cells in human ESC medium or on Matrigel (BD Biosciences)-coated plates in mTeSR medium (STEMCELL Technology). The ESC medium consisted of DMEM/F12 (Invitrogen) supplemented with 20% KnockOut Serum Replacement (Invitrogen), 0.1 mmol/L non-essential amino acids (NEAA, Invitrogen), 1% penicillin/streptomycin (Gibco), 2 mmol/L GlutaMAX (Invitrogen), 55 μmol/L β-mercaptoethanol (Invitrogen), and 10 ng/mL bFGF (Joint Protein Central).

### Plasmid construction

Guide RNA (gRNA) was designed with http://crispr.mit.edu. The gRNAs were cloned into the pCAG-mCherry-gRNA vector (Addgene #87110). For the expression of Cas9 and GFP (Cas9-2A-GFP), the pCAG-1BPNLS-Cas9-1BPNLS-2AGFP plasmid (Addgene #87109) was used (Suzuki et al., 2016). The sequences for the gRNA target and ssODN used to repair mutant alleles are as follows: Exon 4-gRNA: GGATCACGCCAGTCTGGAGTAGG. *ERCC6*-ssODN, 5′-CTAAAGAGACACCCTCCACTGACTACAGGCATCAGGCATCAATTCAAGAACACAGAGAAACTGCTCCTAGCATCCTCACCTGCATCCTCtTCCAGACTGGCGTGATCTAGTTCAATTTTCACCTCTG-3′.

### Targeted gene correction in CS-iPSCs via the CRISPR/Cas9 system

CRISPR/Cas9-mediated gene correction of *ERCC6* mutation was performed as previously described with some modifications (Peters et al., 2008). Briefly, 5 × 10^6^ iPSCs were resuspended in 100 μL of Opti-MEM (Gibco) supplemented with 8 μg of Cas9-2A-GFP, 4 μg of gRNA-mCherry, and 8 μg of ssODN. After electroporation, the cells were cultured on Matrigel-coated plates in mTeSR medium. At forty-eight hours after electroporation, mCherry^+^/GFP^+^ cells were collected by FACS and replated onto MEF feeder cells. Two weeks later, the iPSC clones were picked and identified by genomic DNA PCR and sequencing. The primers used are listed in Table S1.

### MSC generation and characterization

The differentiation of CS-iPSCs and GC-iPSCs into MSCs was performed as previously described (Zhang et al., 2015; Pan et al., 2016; Wang et al., 2018b). Briefly, embryoid bodies were plated onto Matrigel-coated plates in differentiation medium (αMEM (Invitrogen) supplemented with 10% FBS (Gemcell), 10 ng/mL bFGF (Joint Protein Central, JPC), 5 ng/mL TGFβ (Human Zyme), 0.1 mmol/L NEAA (Gibco) and 1% penicillin/ streptomycin (Gibco)). The differentiated cells were then subjected to FACS to purify the CD73/CD90/CD105 (MSC-specific surface markers) triple-positive MSCs. The purified MSCs were then cultured in αMEM medium supplemented with 10% FBS, 1 ng/mL bFGF, 1% penicillin/streptomycin, and 0.1 mmol/L NEAA.

Clinical-grade MSC differentiation and culture were performed in the cGMP level cell culture facility (Clinical-grade Stem Cell Research Center, Peking University Third Hospital) following the cGMP compliance guidelines. First, differentiation of iPSCs into MSCs was achieved using process similar to that used for general MSCs except prepared in a xeno-free and serum-free condition. Briefly, embryoid bodies were plated onto vitronectin (Gibco, A14700)-coated plates in differentiation medium (BM MSC medium (Dakewe, DKW34-BM20500) supplemented with 5% serum replacement (Helios, GMP grade, HPCFDCGL50), 5 ng/mL TGFβ (Human Zyme), 6 ng/mL bFGF (Joint Protein Central, JPC), 10 ng/mL EGF (Joint Protein Central, JPC), 10 ng/mL PDGF (Joint Protein Central, JPC) and 1% penicillin/streptomycin (Gibco)). Next, the differentiated cells were subjected to FACS to purify the CD73/CD90/CD105 triple-positive MSCs. The purified MSCs were then cultured in BM MSC medium supplemented with 5% serum replacement and 1% penicillin/streptomycin.

The differentiation potential of the MSCs towards chondrocytes, osteoblasts and adipocytes was evaluated by staining with Alcian blue (chondrogenesis), von Kossa (osteogenesis) and an oil red O (adipogenesis) kit (IHC World) after differentiation of the indicated lineage, as previously described (Zhang et al., 2015; Pan et al., 2016; Wang et al., 2018b).

### Sterility and pathogen testing of MSCs generated under a cGMP-compliant condition

The conditioned medium of GC-MSCs was collected for the following test. Cell debris in the conditioned medium was removed by centrifugation at 12,000 rpm and 4 °C for 5 min. In addition, the cell culture supernatant was immediately assayed. For CMV, HAV, HCV and HIV-1 ELISA detection, the optical density (O.D.) value for each sample was determined using a microplate reader set to 450 nm (OD_450_). The duplicate readings for each standard, control, and experimental sample were averaged, and the average zero standard O.D. was subtracted.

- Mycoplasma detection

Mycoplasma in the supernatant of the conditioned medium was detected by PCR. The primer sequences are listed in Table S1.

b. Endotoxin detection

Endotoxin in the supernatant of the conditioned medium was detected with the ToxinSensor Gel Clot Endotoxin Assay Kit (GenScript, Cat. No. L00351) according to the manufacturer’s protocol. Briefly, 100 μL of the supernatants from the positive control (PC), negative control (NC) or experimental samples was transferred to the LAL reagent. The vials were capped and mixed thoroughly. All vials were placed in the incubation rack and incubated at 37 °C for 60 min. Then, the vials were inverted and checked to determine whether a gel was formed. The formation of the gel was considered endotoxin positive. The endotoxin level in the positive sample was higher than 0.25 EU/mL.

c. CMV detection

CMV IgM in the conditioned medium was detected by ELISA (MEDSON) according to the manufacturer’s instructions. Briefly, 100 μL of the supernatants from the PC, NC or experimental samples was pipetted onto the microplate. After incubation with antigen and conjugate solution, the absorbance of the samples was determined at 450 nm. The test results are interpreted as a ratio of the sample (S) OD450 nm and the cut-off (Co) value (S/Co) according to the following standard: S/Co < 1.0 was considered negative; S/Co > 1.2 was considered positive. Co = NC + 0.25.

d. HAV detection

HAV IgM and IgG in the conditioned medium were detected by ELISA (DIA. PRO) following the manufacturer’s protocol. Briefly, 100 μL of the supernatants from the PC, NC or experimental samples was pipetted onto the microplate. After incubation with antigen and conjugate solution, the absorbance of the samples was determined at 450 nm. The test results are interpreted as the ratio of the cut-off value to the sample OD_450_ (Co/S) according to the following standard: Co/S < 0.9 was considered negative; Co/S > 1.1 was considered positive. Co = (NC + PC) / 3.

e. HCV detection

HCV IgM and IgG in the conditioned medium were detected by ELISA (DIA. PRO) according to the manufacturer’s guidelines. First, 100 μL of the supernatants from the PC, NC or experimental samples was pipetted onto the microplate. After incubation with antigen and conjugate solution, the test results are interpreted as the ratio of OD_450_ of the sample to the cut-off value (S/Co) according to the following standard: S/Co < 0.9 was considered negative; S/Co > 1.1 was considered positive. Co = NC + 0.35.

f. HIV-1 detection

HIV-1 Gap p24 in the conditioned medium was detected by ELISA (R&D SYSTEMS) according to the manufacturer’s protocol. Briefly, 100 μL of the supernatants from the standard, control or experimental samples was pipetted onto the microplate. After incubation with conjugate solution, the concentration of each sample was calculated by OD_450_. The minimum detectable dose of HIV-1 Gag p24 ranged from 0.24–3.25 pg/mL.

g. Febrile pathogen detection

Pathogens in the conditioned medium were detected by the Febrile Antigens Kit (Rapid Labs). Briefly, 80 μL of the supernatants from the PC, NC or experimental samples was dispensed onto a 3 cm diameter circle. One drop of the antigen suspension was added to the sample. The reaction mixture was mixed well using a stirring stick, and the slide was rocked gently by hand for 1 min. The slides were immediately observed under suitable light for any degree of agglutination. Nonreactive: smooth suspension with no visible agglutination, as shown by the NC. Reactive: any degree of agglutination visible macroscopically.

### NSC generation and characterization

NSC differentiation was conducted as previously described (Liu et al., 2012; Duan et al., 2015). In brief, iPSCs cultured on MEF feeder cells were differentiated with NIM-1 medium [50% Advanced DMEM/F12 (Invitrogen), 50% Neurobasal Medium (Invitrogen), 1× N2 Supplement (Invitrogen), 1× B27 Supplement (Invitrogen), 4 µmol/L CHIR99021 (Cellagentech), 3 µmol/L SB431542 (Cellagentech), 10 ng/mL human leukemia inhibitory factor (hLIF, Millipore), 2 µmol/L dorsomorphin (Sigma), 0.1 µmol/L Compound E (EMD Chemicals Inc.) and 2 mmol/L GlutaMAX (Invitrogen)]. Two days later, the medium was changed to NIM-2 medium (50% Advanced DMEM/F12, 50% Neurobasal Medium, 1× N2 Supplement, 1× B27 Supplement, 4 µmol/L CHIR99021, 3 µmol/L SB431542, 10 ng/mL hLIF, 0.1 µmol/L Compound E and 2 mmol/L GlutaMAX) for five more days. The NSCs were then generated and further cultured in NSC maintenance medium containing 50% Neurobasal Medium, 50% Advanced DMEM/F12, 1× N2 Supplement, 1× B27 Supplement, 2 mmol/L GlutaMAX, 3 μmol/L CHIR99021, 2 μmol/L SB431542 and 10 ng/mL hLIF.

### Animal experiments

All animal experiments performed in this study were approved by the Chinese Academy of Science Institutional Animal Care and Use Committee. For the teratoma formation assay, 6-week-old male NOD-SCID mice were injected subcutaneously with 3 × 10^6^ CS-iPSCs or GC-iPSCs in a Matrigel/mTeSR solution, as previously described (Zhang et al., 2015). Teratomas with a size of approximately 10 mm in diameter were collected and subjected to immunostaining. For the MSC *in vivo* imaging assay, 10^6^ CS-MSCs or GC-MSCs expressing luciferase were transplanted into the TA muscle of 6-week-old male nude mice. The grafted cells were imaged with an IVIS spectrum imaging system (XENOGEN, Caliper) by detecting luciferase activity. To evaluate the potential tumorigenesis risk of GC-MSCs *in vivo*, a subcutaneous injection of GC-MSCs was performed in NSG mice. Human ESC (line H9) and U2-OS osteosarcoma cell lines were also implanted independently as positive controls. Fat pad transplantation was performed as previously described (Yu et al., 2016; Geng et al., 2018). CS-MSCs or GC-MSCs (1.5 × 10^5^) were freshly collected and resuspended in Matrigel mixture containing 50% Matrigel, 20% FBS in PBS, and 0.01% Trypan Blue (Sigma). The mixture was then injected into the fat pads of 3-week-old female NOD-SCID mice. Four weeks later, the fat pads were harvested for measuring MSC-derived vessel regeneration by immunofluorescence staining.

### **Senescence**-**associated β**-**galactosidase (SA**-**β**-**Gal) staining assay**

SA-β-Gal staining was performed according to a previously described method (Debacq-Chainiaux et al., 2009; Zhang et al., 2015; Pan et al., 2016; Geng et al., 2018; Wang et al., 2018b). Each experiment was performed in three independent replicates.

### Clonal expansion assay

Approximately 2000 cells were seeded into each well of 12-well plates and cultured for 2 weeks. Then, the cells were stained with 0.2% crystal violet, and the intensity of the crystal violet staining was quantified by ImageJ software. Each experiment was performed in three independent replicates.

### **RT**-**qPCR**

Total RNA was extracted with TRIzol reagent (Invitrogen), and 2 μg of total RNA was used for cDNA synthesis using a reverse transcription master mix (Promega). Quantitative real-time PCR was conducted with the iTaq Universal SYBR Green Super Mix (Bio-Rad) with the CFX384 Real-Time PCR system (Bio-Rad). All data were normalized to the 18S rRNA transcript and calculated using the ΔΔCq method. All RT-qPCR primer pairs are listed in Table S1.

### Western blot

Western blot was performed as previously described (Wang et al., 2015, 2016). Briefly, protein quantification was conducted using a BCA Kit. Protein lysates were subjected to SDS-PAGE and subsequently electrotransferred to a polyvinylidene fluoride membrane (Millipore). The membrane was incubated with the indicated primary antibodies overnight at 4 °C and HRP-conjugated secondary antibodies for 1 h at room temperate (RT), followed by visualization using the ChemiDoc XRS system (Bio-Rad). Quantification was performed with ImageJ software.

### Immunofluorescence

Immunofluorescence was conducted as previously described (Wang et al., 2016). Briefly, the cells were fixed with 4% paraformaldehyde for 25 min, permeabilized with Triton X-100 (0.3% in PBS) for 25 min, incubated with blocking buffer (10% donkey serum in PBS) for 1 h at RT, and stained with primary antibodies overnight at 4 °C. Then, the cells were incubated with secondary antibodies for 1 h at RT. Hoechst 33342 (Invitrogen) was used to stain nuclear DNA.

### Analysis of apoptosis by flow cytometry

A FACS-based apoptosis analysis was performed as previously described (Fu et al., 2016; Pan et al., 2016). For ROS measurement, cells were collected and incubated with 1 μmol/L H2DCFDA for 30 min using ROS Detection Reagents (Molecular Probes, C6827). The cells were later analysed using the BD LSRFortessa cell analyser.

### RNA sequencing library construction

Total RNA for each sample was extracted using the RNeasy Mini Kit (Qiagen) according to the manufacturer’s instructions. After quantification of the RNA by a fragment analyzer (Advanced Analytical), RNA sequencing libraries were constructed using the TruSeq RNA Sample Preparation Kit (Illumina) according to the manufacturer’s protocols. Paired-end sequencing was performed using Illumina Hiseq X Ten platform.

### RNA sequencing data processing

RNA-seq data processing was performed as previously described (Zhang et al., 2015, 2019; Geng et al., 2018; Wang et al., 2018a; Ling et al., 2019). In brief, sequencing reads were trimmed and mapped to the *H. sapiens* reference genome (hg19) with HISAT2 software (v2.0.4) (Kim et al., 2015). HTSeq (v0.10.0) was used to determine the transcriptional expression level of each gene (Anders et al., 2015). Differentially expressed genes (DEGs) were computed at a cut-off adjusted *P* value (Benjamini-Hochberg) less than 0.05 and |Log_2_(fold change)| more than 1 using DESeq2 (Love et al., 2014). Pearson’s correlation coefficient (*R*) and the Euclidian distance were calculated using *R* to evaluate the correlation between the replicates of each sample, which were based on Log_2_(FPKM + 1). PCA was also performed using *R* based on Log_2_(FPKM + 1). Gene ontology (GO) enrichment analysis was computed by Metascape (Tripathi et al., 2015). The enrichment networks were visualized using Cytoscape (Shannon et al., 2003). Protein-protein interaction networks of overlapping genes were drawn based on the search tool for the retrieval of interacting genes (STRING) database (Szklarczyk et al., 2017). The aging-associated genes were obtained from the human aging genomic resources (HAGR) database (Tacutu et al., 2013).

### DNA extraction, library construction and sequencing

Genomic DNA was extracted from each sample using the QIAamp® DNA Mini Kit (Qiagen), according to the manufacturer’s protocol. DNA was randomly fragmented into ~300 bp lengths using a Covaris ultrasonic processor. DNA libraries were prepared with the NEBNext® Ultra^TM^ DNA library Prep Kit (Illumina) and quantified using a Qubit 2.0 Fluorometer (Life Technologies). The insert sizes of the fragments in the libraries were determined by the Agilent Bioanalyzer 2100. Paired-end sequencing was performed using the Illumina HiSeq X Ten platform.

### Bioinformatics analyses of copy number variations, single-nucleotide variants and off-target sites

The pipeline of whole genome sequencing data processing used in this study has been described previously (Zhang et al., 2018). In brief, sequencing data were mapped to the *H*. *sapiens* reference genome (hg19) without repeat regions using the Burrows-Wheeler Aligner (BWA, version 0.7.17) (Li and Durbin, 2009). The genomic coverage for each 500 kb bin window was calculated and normalized by the average sequencing depth. The copy number variation (CNV) scatterplot was drawn by ggplot2. For the single-nucleotide variant (SNV) analysis, the read base sites with an incorrect base probability >0.001 were masked with N, and base distribution for each chromosomal location was calculated by pysamstats (version 1.0.1) (https://github.com/alimanfoo/pysamstats). The heterozygosity of each site was defined as the percentage of the second enriched base depth. SNV sites were defined by base heterozygosity (0%–30%). Potential indel sites were extracted with pysamstats (version 1.0.1) under default setting. Then indel sites were screened with sites existing in CS-iPSC genomic sequencing datasets, repeats and low-complexity regions annotated by RepeatMasker (db20170127), indel-type SNPs in humans and homopolymers. Simultaneously, 2034 off-target sites with no more than five mismatched sites were identified by Cas-OFFinder (Bae et al., 2014). None of these regions included indel sites identified by whole genome sequencing.

### Statistical analysis

All results are presented as the mean ± SEM or mean ± SD. The data were statistically analysed using a two-tailed Student’s *t*-test to compare differences between treatments assuming equal variance with PRISM software (GraphPad 5 Software). *P* values <0.05, <0.01, and <0.001 were considered statistically significant (*, **, and ***, respectively).

### Accession numbers

The sequencing data have been deposited in the NCBI Gene Expression Omnibus (GEO) under the accession number GSE124208, NCBI Sequence Read Archive under accession number SRP174074.

## Electronic supplementary material

Below is the link to the electronic supplementary material.

Supplementary material 1 (PDF 3822 kb)

Supplementary material 2 (XLSX 13 kb)
