# Correction to: Rescue of premature aging defects in Cockayne syndrome stem cells by CRISPR/Cas9-mediated gene correction

**DOI:** 10.1007/s13238-021-00901-3

**Published:** 2022-01-15

**Authors:** Si Wang, Zheying Min, Qianzhao Ji, Lingling Geng, Yao Su, Zunpeng Liu, Huifang Hu, Lixia Wang, Weiqi Zhang, Keiichiro Suzuiki, Yu Huang, Puyao Zhang, Tie-Shan Tang, Jing Qu, Yang Yu, Guang-Hui Liu, Jie Qiao

**Affiliations:** 1 Department of Obstetrics and Gynecology, Center for Reproductive Medicine, Peking University Third Hospital, Beijing, 100191 China; 2 National Laboratory of Biomacromolecules, CAS Center for Excellence in Biomacromolecules, Institute of Biophysics, Chinese Academy of Sciences, Beijing, 100101 China; 3 State Key Laboratory of Stem Cell and Reproductive Biology, Institute of Zoology, Chinese Academy of Sciences, Beijing, 100101 China; 4 University of Chinese Academy of Sciences, Beijing, 100049 China; 5 Advanced Innovation Center for Human Brain Protection, National Clinical Research Center for Geriatric Disorders, Xuanwu Hospital Capital Medical University, Beijing, 100053 China; 6 Institute for Stem cell and Regeneration, Chinese Academy of Sciences, Beijing, 100101 China; 7 Key Laboratory of Genomic and Precision Medicine, Beijing Institute of Genomics, Chinese Academy of Sciences, Beijing, 100101 China; 8 Beijing Institute for Brain Disorders, Beijing, 100069 China; 9 Institute for Advanced Co-Creation Studies, Osaka University, Osaka, 560-8531 Japan; 10 Graduate School of Engineering Science, Osaka University, Osaka, 560-8531 Japan; 11 Department of Medical Genetics, School of Basic Medical Sciences, Peking University Health Science Center, Beijing, 100191 China; 12 State Key Laboratory of Membrane Biology, Institute of Zoology, Chinese Academy of Sciences, Beijing, 100101 China; 13 Peking-Tsinghua Center for Life Sciences, Academy for Advanced Interdisciplinary Studies, Peking University, Beijing, 100871 China

## Correction to: Protein Cell 10.1007/s13238-019-0623-2

In Fig. 7C, we used the ERCC6^mut^-iPSCs (CS-iPSCs) as NANOG-positive control pluripotent cells in the upper panels. However, these cells were inadvertently labeled as ERCC6^GC^-iPSCs. In the revised version of Fig. 7C, we have updated the high-quality images along with the corrected mark. In addition, we have also made corresponding changes in the figure legend.

**Figure 7:**
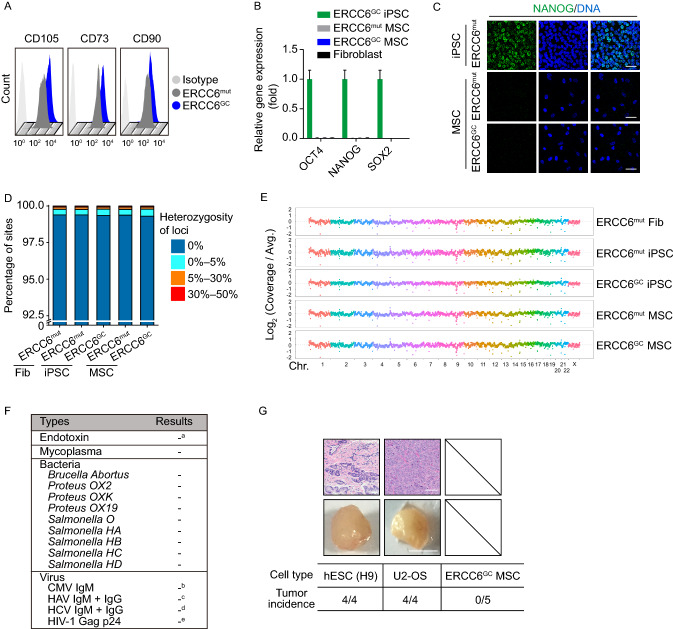
**Safety analysis of gene-corrected CS-MSCs obtained under a cGMP-compliant condition.** (A) FACS analysis indicated the expression of the cell surface markers CD73, CD90 and CD105 in CS-MSCs and GC-MSCs. (B) RT-qPCR analysis of the expression of pluripotency markers OCT4, NANOG, and SOX2 in CS-MSCs and GC-MSCs. GC-iPSCs and CS-fibroblasts were used as positive and negative controls, respectively. Data are presented as the mean ± SEM, *n* = 3. (C) Immunostaining of the pluripotency marker NANOG in CS-MSCs and GC-MSCs. CS-iPSCs were used as a positive control, Scale bar, 50 μm. (D) Whole-genome sequencing of single-nucleotide variants (SNVs) in CS-fibroblasts, CS-iPSCs, GC-iPSCs, CS-MSCs and GC-MSCs. Sites with a heterozygosity percentage ranging between 0% and 30% were considered as SNV sites, and sites with a heterozygosity of >30% were considered as single-nucleotide polymorphisms (SNPs). (E) Whole-genome sequencing of copy number variations (CNVs) in CS-fibroblasts, CS-iPSCs, GC-iPSCs, CS-MSCs and GC-MSCs. Each point represents normalized coverage depth of each 500-kb genomic region of each chromosome. (F) Sterility and pathogen testing of the conditioned medium of GC-MSCs. ^a^ Endotoxin was identified as negative when the concentration was < 0.25 EU/mL. ^b^ CMV was identified as negative when the ratio of the OD450 value of sample to the cutoff value (S/Co) was < 1.0. ^c^ HAV was identified as negative when the ratio of the cut-off value to the OD450 nm value of the sample (Co/S) was < 0.9. ^d^ HCV was identified as negative when the ratio of the OD450 value of the sample to the cut-off value (S/Co) was < 0.9. ^e^ HIV-1 was identified as negative when the concentration = 0 pg/mL. (G) Evaluation of the potential tumorigenesis risk of GC-MSCs *in vivo*. A subcutaneous injection of GC-MSCs was performed in immune-deficient mice. Human ESC (line H9) and U2-OS osteosarcoma cell lines were also implanted independently as positive controls. Representative images in the lower panel showing the teratoma and tumor formed from positive cells two months after transplantation, Scale bar, 0.5 cm. HE staining of a teratoma and tumor were shown in the upper panel. Scale bar, 100 μm. The *in vivo* tumor-formation incidence of each cell type was calculated. *n* = 4 for each positive cell group, *n* = 5 for the GC-MSC group

