# Supplementary material for: Rescue of premature aging defects in Cockayne syndrome stem cells by CRISPR/Cas9-mediated gene correction
Source: Protein Cell. 2019 Apr 30;11(1):1–22. doi: 10.1007/s13238-019-0623-2 (PMC6949206; doi:10.1007/s13238-019-0623-2)
Supplement: Supplementary file 1 — Supplementary material 1 (PDF 3822 kb) [file 13238_2019_623_MOESM1_ESM.pdf]

Supplementary Figure Legends

Figure S1. Gene expression analyses of CS-iPSCs and gene-corrected CS-iPSCs and their adult stem cell derivatives.

(A) Scatter plots showing the correlation between two replicates for the indicated cell samples. Ctrl: untreated; UV: treatment with UV irradiation; RS: under RS stress; rep: replicate.

(B) Heatmaps showing the pairwise Euclidian distance among the indicated cell samples. Ctrl: untreated; UV: treatment with UV irradiation; RS: under RS stress.

(C) Heatmaps showing the numbers of differentially expressed genes between CS-iPSCs and GC-iPSCs, between CS-MSCs and GC-MSCs, and between CS-NSCs and GC-NSCs. Ctrl: untreated; UV: treatment with UV irradiation; RS: under RS stress. Red represents upregulated genes, and blue represents downregulated genes. “GC” stands for GC cells, and “mut” stands for CS cells.

(D) Principal component analysis (PCA) of the RNA profiles of CS-MSCs and GC-MSCs in the absence or presence of UV irradiation (Ctrl or UV), as well as under RS stress (RS). Each point represents a sample. Data points were computed based on Log_2(FPKM + 1).

(E) Volcano plots showing the differentially expressed genes between UV-treated and untreated MSCs (UV vs. Ctrl, left panel), between MSCs in the presence and MSCs in the absence of RS stress (RS vs. Ctrl, middle panel), and between UV-treated and untreated NSCs (UV vs. Ctrl, right panel). Red represents upregulated genes, and blue represents downregulated genes.

Figure S2. Transcriptome analysis of stress-induced genes in CS and gene-corrected CS adult stem cell derivatives.

(A) Heatmaps showing the upregulated/downregulated genes in CS-MSCs but not in GC-MSCs upon UV treatment (UV vs. Ctrl, left panel) or under RS stress (RS vs. Ctrl, middle panel). Heatmaps in the right panel showing the upregulated/downregulated genes in CS-NSCs but not in GC-NSCs upon UV treatment (UV vs. Ctrl).

(B) Venn diagrams showing the number of overlapping downregulated genes in CS-cells but not in GC-cells. Gene set A indicated the overlapping downregulated genes in CS-cells but not in GC-cells both upon UV irradiation and under RS stress. Gene set B indicated the overlapping downregulated genes in both CS-MSCs and CS-NSCs but not in GC-cells upon UV irradiation.

(C-D) Gene Ontology Biological Process (GO-BP) enrichment analysis of overlapping downregulated genes from gene set A (C) or gene set B (D). The network showing the relationship between different terms is visualized using Cytoscape. Each node

represents an enriched term, each color indicates a cluster consisting of similar terms, and each line represents the correlation between two clusters/terms. The larger the node, the more terms are associated with it. The thicker the line is, the closer relationship between the two clusters/terms.

(E) An interaction network of overlapping genes was constructed using the STRING database. Red represents the overlapping genes from set A and the aging database, blue represents the overlapping genes from set B and the aging database. The solid line represents the interaction between genes from the same gene set, and the dotted line represents the interaction between genes from the different gene sets. The larger the node is, the more genes interact with it.

Figure S3. Analysis of ROS level in iPSCs and evaluation of tri-lineage differentiation potential of MSCs obtained under a cGMP-compliant condition.

(A) Cellular ROS levels of CS-iPSCs and GC-iPSCs were determined by staining with H2DCFDA probe and measured with FACS.

(B) Heatmap showing the expression of genes involved in oxidative stress response in the CS-iPSCs and GC-iPSCs by RT-qPCR. The transcript levels were normalized to CS-iPSCs.

(C) Comparative analysis of the osteogenic, chondrogenic and adipogenic differentiation potential of CS-MSCs and GC-MSCs generated under a cGMP-compliant condition. Von Kossa, Alcian blue, and oil red O staining were used to characterize osteoblasts, chondrocytes, and adipocytes, respectively. Scale bar, 50 μm.

(D) The intensity of von Kossa staining was calculated by ImageJ and compared in the left panel. Data are presented as the mean ± SEM, $n = 3$, * $P < 0.05$. The cross-sectional area of chondrocyte spheres was compared in the middle panel. Data are presented as the mean ± SD, $n = 15$, ** $P < 0.01$. The relative intensity of oil red O was measured and compared in the right panel. Data are presented as the mean ± SEM, $n = 3$, *** $P < 0.001$.

Figure S1

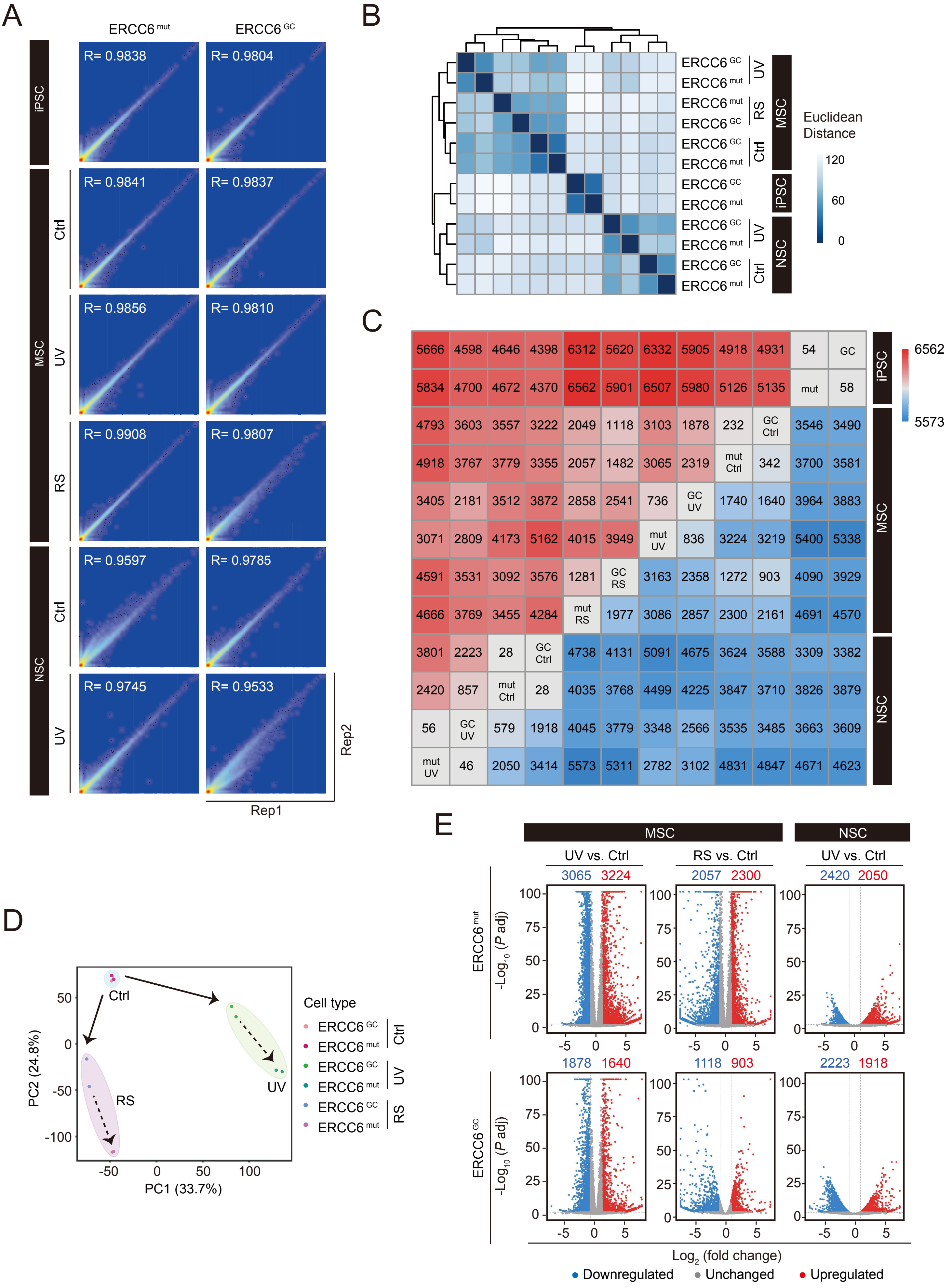
A
ERCC6 mut
ERCC6 GC
iPSC
R= 0.9838
R= 0.9804
MSC
Ctrl
R= 0.9841
R= 0.9837
UV
R= 0.9856
R= 0.9810
RS
R= 0.9908
R= 0.9807
NSC
Ctrl
R= 0.9597
R= 0.9785
UV
R= 0.9745
R= 0.9533
Rep2
Rep1
B
ERCC6 GC
ERCC6 mut
ERCC6 mut
ERCC6 GC
ERCC6 GC
ERCC6 mut
ERCC6 GC
ERCC6 mut
ERCC6 GC
ERCC6 mut
ERCC6 GC
ERCC6 mut
UV
RS
Ctrl
MSC
iPSC
UV
Ctrl
NSC
Euclidean Distance
120
60
0
C
5666 4598 4646 4398 6312 5620 6332 5905 4918 4931 54 GC
5834 4700 4672 4370 6562 5901 6507 5980 5126 5135 mut 58
4793 3603 3557 3222 2049 1118 3103 1878 232 GC Ctrl 3546 3490
4918 3767 3779 3355 2057 1482 3065 2319 mut Ctrl 342 3700 3581
3405 2181 3512 3872 2858 2541 736 GC UV 1740 1640 3964 3883
3071 2809 4173 5162 4015 3949 mut UV 836 3224 3219 5400 5338
4591 3531 3092 3576 1281 GC RS 3163 2358 1272 903 4090 3929
4666 3769 3455 4284 mut RS 1977 3086 2857 2300 2161 4691 4570
3801 2223 28 GC Ctrl 4738 4131 5091 4675 3624 3588 3309 3382
2420 857 mut Ctrl 28 4035 3768 4499 4225 3847 3710 3826 3879
56 GC UV 579 1918 4045 3779 3348 2566 3535 3485 3663 3609
mut UV 46 2050 3414 5573 5311 2782 3102 4831 4847 4671 4623
iPSC
MSC
NSC
6562
5573
D
Ctrl
RS
UV
PC2 (24.8%)
PC1 (33.7%)
50
0
-50
-100
-50
0
50
100
Cell type
ERCC6 GC
ERCC6 mut
ERCC6 GC
ERCC6 mut
ERCC6 GC
ERCC6 mut
Ctrl
UV
RS
E
MSC
NSC
UV vs. Ctrl
RS vs. Ctrl
UV vs. Ctrl
3065 3224
2057 2300
2420 2050
ERCC6 mut
-Log10 (P adj)
100
75
50
25
0
-5 0 5
1878 1640
1118 903
2223 1918
ERCC6 GC
Log2 (fold change)
Downregulated
Unchanged
Upregulated

Figure S2

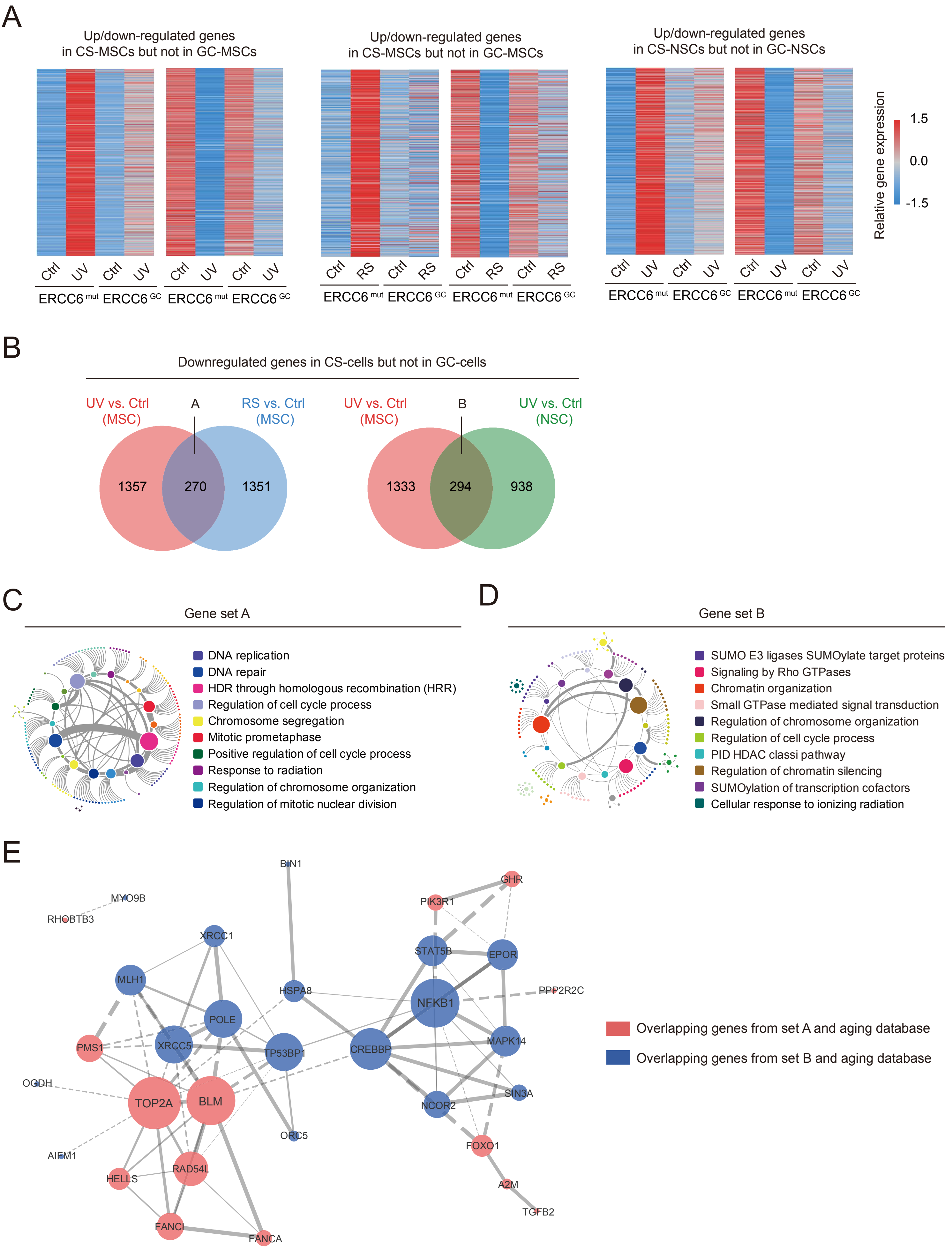
A
Up/down-regulated genes in CS-MSCs but not in GC-MSCs
Up/down-regulated genes in CS-MSCs but not in GC-MSCs
Up/down-regulated genes in CS-NSCs but not in GC-NSCs
Ctrl UV Ctrl UV Ctrl UV Ctrl UV
ERCC6 mut ERCC6 GC ERCC6 mut ERCC6 GC
Ctrl RS Ctrl RS Ctrl RS Ctrl RS
ERCC6 mut ERCC6 GC ERCC6 mut ERCC6 GC
Ctrl UV Ctrl UV Ctrl UV Ctrl UV
ERCC6 mut ERCC6 GC ERCC6 mut ERCC6 GC
Relative gene expression
1.5
0.0
-1.5
B
Downregulated genes in CS-cells but not in GC-cells
UV vs. Ctrl (MSC)
A
RS vs. Ctrl (MSC)
1357
270
1351
UV vs. Ctrl (MSC)
B
UV vs. Ctrl (NSC)
1333
294
938
C
Gene set A
DNA replication
DNA repair
HDR through homologous recombination (HRR)
Regulation of cell cycle process
Chromosome segregation
Mitotic prometaphase
Positive regulation of cell cycle process
Response to radiation
Regulation of chromosome organization
Regulation of mitotic nuclear division
D
Gene set B
SUMO E3 ligases SUMOylate target proteins
Signaling by Rho GTPases
Chromatin organization
Small GTPase mediated signal transduction
Regulation of chromosome organization
Regulation of cell cycle process
PID HDAC classi pathway
Regulation of chromatin silencing
SUMOylation of transcription cofactors
Cellular response to ionizing radiation
E
BIN1
GHR
MYO9B
PIK3R1
RHOBTB3
XRCC1
STAT5B
EPOR
MLH1
HSPA8
PPP2R2C
NFKB1
POLE
PMS1
XRCC5
TP53BP1
CREBBP
MAPK14
OGDH
SIN3A
TOP2A
BLM
NCOR2
ORC5
FOXO1
AIFM1
RAD54L
HELLS
A2M
TGFB2
FANCI
FANCA
Overlapping genes from set A and aging database
Overlapping genes from set B and aging database

Figure S3

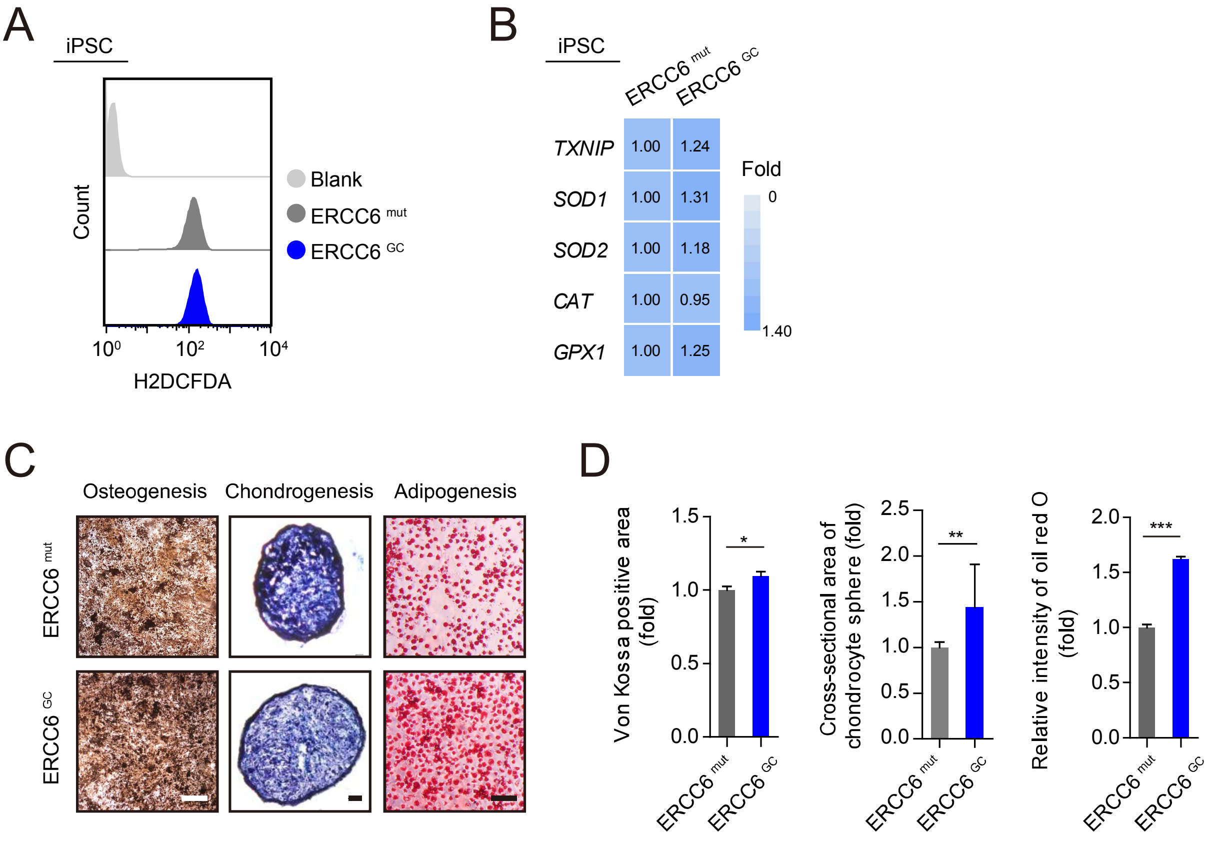

A
iPSC
Count
Blank
ERCC6 mut
ERCC6 GC
10⁰
10²
10⁴
H2DCFDA
B
iPSC
ERCC6 mut
ERCC6 GC
TXNIP 1.00 1.24
SOD1 1.00 1.31
SOD2 1.00 1.18
CAT 1.00 0.95
GPX1 1.00 1.25
Fold
0
1.40
C
Osteogenesis
Chondrogenesis
Adipogenesis
ERCC6 mut
ERCC6 GC
D
Von Kossa positive area (fold)
1.5
1.0
0.5
0.0
*
ERCC6 mut
ERCC6 GC
Cross-sectional area of chondrocyte sphere (fold)
2.5
2.0
1.5
1.0
0.5
0.0
**
ERCC6 mut
ERCC6 GC
Relative intensity of oil red O (fold)
2.0
1.5
1.0
0.5
0.0
***
ERCC6 mut
ERCC6 GC
